# Superior inflammatory response and MASH progression in *Apoe*^-/-^ mice compared to wild-type mice: a comprehensive time-course analysis

**DOI:** 10.3389/fimmu.2026.1895809

**Published:** 2026-07-17

**Authors:** Anja R. Geisler, Nora Meinhardt, Laura Otto, Yvonne Hupfer, Betty Hebecker, Bill J. Perkowski, Markus Werner, Wan-Ting Zhao, Karl-Heinz Herrmann, Marcus Ebert, Sandra Burghoff, Jürgen R. Reichenbach, Norbert Gerdes, Oliver Werz, Anna P. Kipp, Stefan Lorkowski, Maria Witt-Wallert

**Affiliations:** 1 Institute of Nutritional Science, Friedrich Schiller University Jena, Jena, Germany; 2 Institute of Pharmacy, Friedrich Schiller University Jena, Jena, Germany; 3 Medical Physics Group, Institute of Diagnostic and Interventional Radiology, Jena University Hospital, Friedrich Schiller University Jena, Jena, Germany; 4 MVZ Medical Laboratories Dessau Kassel GmbH, Dessau, Germany; 5 Department of Cardiology, Pulmonology, and Vascular Medicine, Medical Faculty and University Hospital, Heinrich Heine University Düsseldorf, Düsseldorf, Germany

**Keywords:** APOE, inflammation, MASLD, metabolic dysfunction, NAFLD

## Abstract

**Background:**

Metabolic dysfunction-associated steatohepatitis (MASH) represents a critical progression of metabolic dysfunction-associated steatotic liver disease (MASLD), characterized by hepatic steatosis, inflammation, and beginning fibrosis. The selection of appropriate animal models is crucial for understanding disease mechanisms and evaluating therapeutic interventions. While wild-type C57BL/6 mice are widely used, hyperlipidemic apolipoprotein E-deficient (*Apoe*^-/-^) mice may offer superior disease modeling capabilities. This study compared the development and progression of diet-induced MASH in *Apoe*^-/-^ mice versus wild-type C57BL/6J mice over multiple time points, with a focus on inflammatory responses and histological changes.

**Methods:**

Male wild-type C57BL/6J and male *Apoe*^-/-^ mice were fed either standard chow diet or high-fat/high-fructose (Hf/Hf) diet for 8, 12, 16, or 24 weeks. Comprehensive analyses included histological evaluation, MRS based fat quantification, immunohistochemistry, serum biochemistry, cytokine profiling, trace element analysis, oxylipin profiling by UPLC-MS/MS, intestinal barrier function assessment, and gene expression analysis.

**Results:**

*Apoe*^-/-^ mice showed markedly accelerated MASH development compared to wild-type mice. Notably, *Apoe*^-/-^ mice fed Hf/Hf diet for 12 weeks exhibited substantially greater inflammatory infiltration than wild-type mice even after 24 weeks of Hf/Hf diet. This enhanced inflammatory response was accompanied by increased infiltration of macrophages (CD68^+^ cells), elevated levels of pro-inflammatory cytokines, altered trace element homeostasis, and dysregulated oxylipin profiles favoring pro-inflammatory mediators. There was also evidence of compromised intestinal barrier function and more pronounced histological features of MASH, including steatosis, lobular inflammation and hepatocyte ballooning.

**Conclusion:**

*Apoe*^-/-^ mice represent a superior model for MASH research, demonstrating accelerated disease progression and enhanced inflammatory responses compared to the wild-type mice. The rapid and robust inflammatory phenotype in *Apoe*^-/-^ mice provides a more efficient and clinically relevant model for investigating MASH pathogenesis and testing therapeutic interventions.

## 1 Introduction

Metabolic dysfunction-associated steatotic liver disease (MASLD) affects an estimated 1.3 billion individuals worldwide in 2023, representing a substantial increase of over 140% compared to 1990 (1). The progressive form, metabolic dysfunction-associated steatohepatitis (MASH), is characterized by hepatic steatosis accompanied by inflammation and hepatocellular injury, with potential progression to advanced fibrosis, cirrhosis, and hepatocellular carcinoma (2). The pathogenesis of MASH involves complex interactions between metabolic dysfunction, lipotoxicity, oxidative stress, and inflammatory pathways (3). Key mechanistic features include dysregulated lipid metabolism, mitochondrial dysfunction, endoplasmic reticulum (ER) stress, and activation of inflammatory cascades involving both innate and adaptive immune responses (4). Understanding these mechanisms requires robust animal models that accurately recapitulate the human disease phenotype.

Currently, various mouse models are used in MASH research, with C57BL/6 mice being a commonly used genotype due to their susceptibility to diet-induced obesity and hepatic steatosis (5, 6). However, wild-type C57BL/6 mice often require prolonged feeding periods or additional (e.g. chemical) interventions to develop hepatic inflammation and fibrosis, limiting their usability for mechanistic studies and therapeutic testing (7). Apolipoprotein E-deficient (*Apoe*^-/-^) mice, originally developed as a hyperlipidemic model of atherosclerosis, have emerged as a potential alternative in MASH research (8, 9). Apolipoprotein E (APOE) plays crucial roles in lipid metabolism, and its deficiency leads to severe dyslipidemia and enhanced inflammatory responses (10). These mice spontaneously develop atherosclerosis on standard chow diet and show exaggerated responses to dietary challenges (11). Studies suggest that *Apoe*^-/-^ mice may develop more severe and rapid MASH phenotypes compared to wild-type mice, potentially offering advantages for research applications (9).

The selection of appropriate animal models is critical for the success in translational research. An ideal MASH model should exhibit: (i) rapid and reproducible disease development, (ii) histological features resembling human disease, (iii) robust inflammatory responses, (iv) progression to fibrosis, and (v) responsiveness to therapeutic interventions (12). While wild-type C57BL/6 mice fulfill many of these criteria, the prolonged feeding period required to induce a robust phenotype necessitates more frequent compound administration during compound testing. This results in increased handling-related stress and animal burden, which may confound metabolic and inflammatory readouts and limit the usability of the model.

The Western dietary pattern is characterized by the persistent overconsumption of energy-dense, highly processed foods and added sugar, alongside a low intake of dietary fiber and polyunsaturated fats. These features are major drivers of lifestyle-related noncommunicable diseases, such as obesity, type 2 diabetes mellitus, cardiovascular diseases, and steatotic liver diseases. In particular, the high intake of sugar, especially sugar-sweetened beverages and fructose-sweetened food, are repeatedly implicated in promoting type 2 diabetes mellitus, cardiovascular diseases, as well as hepatic *de novo* lipogenesis and hepatic fat deposition (13–15). In addition high saturated fats, trans fats and excessive dietary cholesterol promote hepatic lipid accumulation, lipotoxicity and adverse lipid profiles (14, 16, 17).

To meet these metabolic challenges, a mouse model that closely mimics this dietary pattern was used, by applying a high-fat/high-fructose (Hf/Hf) diet to study its metabolic and hepatic consequences. Despite the potential advantages of *Apoe*^-/-^ mice for MASH research, comprehensive time-course comparisons with wild-type C57BL/6J models are lacking. Understanding the temporal progression of disease features is essential for optimizing experimental design and for better translating findings to the human condition. Therefore, this study aimed to conduct a systematic and comprehensive comparison of MASH development and progression in *Apoe*^-/-^ mice compared to wild-type C57BL/6J mice over multiple time points (8, 12, 16, and 24 weeks) using a standardized Hf/Hf diet. It was hypothesized that *Apoe*^-/-^ mice would exhibit an accelerated and more severe MASH phenotype, particularly with respect to inflammatory responses, compared to the wild-type C57BL/6J model.

## 2 Methods

### 2.1 Animal experiments

Male wild-type C57BL/6J mice (C57BL/6JRj; 8–10 weeks; Janvier Labs, France) and male *Apoe*^-/-^ mice, with C57BL6J background (B6.129P2-Apoe^tm 1Unc^/J; 10–17 weeks; kindly provided by Norbert Gerdes, Heinrich Heine University Düsseldorf, Germany), were housed in a specific and opportunistic pathogen free ( SOPF) barrier facility. The environment was maintained at 22 ± 2 °C, relative humidity 55% ± 10%, a 12 h light/12 h dark cycle. Mice had free access to food and water. The mice were provided with a range of enrichments designed to stimulate their natural behaviors, these included paper rolls, gnawing bricks and paper houses.

The mice were randomly divided into two groups: control diet-fed (regular chow; Cat. No. V1534-300, Ssniff, Soest, Germany) and Hf/Hf diet containing 45 MJ% fat, 42 MJ% carbohydrates (with 25% fructose w/w) and 1% cholesterol (Cat. No. S8756-E722, Ssniff) (see **Supplementary Table 1**). Animals were fed the respective diets for 8, 12, 16, or 24 weeks. All animal procedures were approved by the Thuringian State Office for Consumer Protection (TLV, Erfurt, Germany), under the file reference FSU-23-004.

### 2.2 Histology

All staining was performed using an automated stainer (Gemini AS, Epredia, Dreieich, Germany). Paraffin-embedded, formalin-fixed liver was cut into 6 µm sections using a microtome (RM2245, Leica, Nußloch, Germany). Slides were deparaffinated in Histol (2×10 min; Carl Roth, Karlsruhe, Germany) and rehydrated in a graded ethanol series (100%, 95%, 85%, 70%, 40%; 10 min) followed by running distilled water (20 min). Hematoxylin and eosin (H&E) staining: Sections were stained with Gill’s hematoxylin No. 2 (90 s; Merck, Darmstadt, Germany), rinsed in tap water (2×10 s), and placed in running distilled water (10 min). Slides were then dehydrated in 70% (7 min) and 85% ethanol (7 min), counterstained with eosin Y (1 min; Carl Roth), followed by 95% (20 s) and 100% ethanol (20 s), cleared in Histol (2×10 min), and mounted. Picro Sirius Red (PSR) staining: Sections were immersed in 0.1% P SR solution (0.5 g Direct red 80, Merck, in 500 ml picric acid, Merck) for 60 min, rinsed in 0.5% acetic acid/distilled water, washed in running distilled water (5 min), dehydrated in absolute ethanol (3×5 min), cleared in Histol (2×5 min), and mounted. Immunohistological (IHC) staining: Paraffin-embedded sections were deparaffinized in Histol (2×5 min) and rehydrated through a graded ethanol series (100% 2×3 min, 96% 3 min, 70% 3 min), followed by distilled water (2×3 min). Antigen retrieval was performed in Tris/EDTA buffer (10mM/1mM; pH 9.0) in a water bath at 95 °C for 25 min, followed by slow cooling at room temperature (RT) for 20 min. Slides were washed in phosphate-buffered saline with 0.1% (w/v) Tween detergent (PBST) (2×5 min). Endogenous peroxidase activity was blocked by flooding slides with 3% H_2_O_2_/Methanol (30 min; RT). Slides were washed in PBST (2×5 min) and circled with a wax pen to contain reagents. Blocking steps were performed sequentially: avidin block (15 min; Abcam, Cambridge, U.K.), washing in PBST (5 min), biotin block (15 min; Abcam), washing in PBST (5 min), and 5% bovine serum albumin (BSA) in phosphate-buffered saline (PBS) (30 min; Carl Roth). Primary antibodies, diluted in 0.5% BSA in PBS, were applied overnight at 4 °C in a humidified chamber. The following antibodies were used: cluster of differentiation 68 (CD68), 1:250, Abcam, Cat. No. ab283654; IL-1β, 1:100, Abcam, Cat. No. ab315084; 3-nitrotyrosine (3-NT), 1:100, Abcam, Cat. No. ab42789. Slides were washed in PBST (3×5 min), followed by incubation with secondary antibody (30 min; Vectastain Elite ABC Universal plus Kit, Biozol, Eching, Germany), then washed in PBST (3×5 min). ABC reagent (30 min, RT, same kit) was applied, followed by washing in PBST (2×5 min). Diaminobenzidine (DAB) substrate solution was applied, and the reaction was monitored under a microscope. The reaction was stopped by immersion in distilled water once a brown color developed. Sections were rinsed in distilled water (2×5 min), counterstained with Gill’s hematoxylin No. 2 (45 s), rinsed in running tap water (5 min), dehydrated through 95% (3 min) and 100% ethanol (2×3 min) cleared in Histol (2×5 min), and mounted. Frozen liver was mounted in O.C.T. (Science Services, Munich, Germany) cut into 6 µm sections using a cryostat (CM1520, Leica) thawed for 30 min and fixed in Roti Histofix (4 min; Carl Roth). Oil Red O (ORO) staining: Sections were rinsed in 60% isopropanol (30 s) and stained with ORO (Merck; stock solution: 1 g/100 ml isopropanol, filtered; working solution: 3:2 dilution with distilled water) for 15 min. Differentiation was performed by dipping slides three times in 60% isopropanol, followed by rinsing in distilled water (2 min). Counterstaining was carried out with Gill’s No. 2 hematoxylin (30 s), followed by rinsing in tap water (3 min) and running distilled water (3 min), before mounting. H&E staining: Sections were immersed in 70% ethanol (2×30 s) and rinsed in distilled water (10 min). Slides were stained with Gill’s hematoxylin No. 2 (2 min), rinsed in tap water (5 min), and differentiated in 1% HCl/70% ethanol (3 s). After washing in tap water (5 min) and distilled water (2×5 min), sections were counterstained with eosin Y (1%, 1 min), followed by washing in tap water (2×5 min) and distilled water (2×5 min). Sections were dehydrated in 100% isopropanol (2 min) and mounted.

Images were acquired with a BZ-X800 microscope (Keyence, Frankfurt/Main, Germany), and quantitative analysis was carried out using ImageJ software. For each staining and animal, at least two representative liver sections were analyzed, and a total of ten regions were evaluated. As described by Kleiner et al., Nonalcoholic fatty liver disease activity score (NAS) was determined based on steatosis (0–3), lobular inflammation (0–3), and hepatocellular ballooning (0–2) (18). Steatotic areas were quantified from 10× magnification images following conversion to 8-bit grayscale and threshold-based segmentation. Lipid droplets were identified according to pixel intensity and particle size using automated batch processing with custom ImageJ macros. PSR-positive areas were quantified from images acquired at 10× magnification, as previously described (19). Briefly, images were converted into hue and saturation channels, and low-saturation background pixels were excluded by threshold-based masking. Red and yellow collagen-associated signals were subsequently identified using predefined hue ranges and quantified by automated pixel counting. Fibrotic area was calculated as the fraction of red-positive pixels relative to the total collagen-positive area. ORO-positive areas were quantified from images acquired at 20× magnification using automated batch processing. Red-stained regions were isolated by converting images into hue, saturation, and brightness channels, followed by threshold-based masking of saturation and hue channels and mask combination. Quantification was performed after 8-bit conversion and threshold-based segmentation. Immunohistochemical staining was quantified from 20× magnification images using the “Colour Deconvolution” plugin (H-DAB setting) to separate hematoxylin and DAB signals. DAB images were converted to 8-bit grayscale, thresholded, and transformed into binary masks. DAB-positive areas were quantified using custom ImageJ macros in batch mode. Generated masks were visually inspected to verify accurate detection of positively stained areas. All histological alterations and scorings were evaluated in a blinded manner to minimize observer bias.

### 2.3 Serum parameters and enzyme activities

Serum cholesterol, aspartate aminotransferase (AST) and alanine aminotransferase (ALT) were quantified by an automated analyzer (Cobas, Roche Diagnostics, Rotkreuz, Switzerland) with the appropriate commercially available reagent kits. Serum amyloid A (SAA) was determined using ELISA (Thermo Fisher Scientific, Cat. No. KMA0021, Darmstadt, Germany.), according to the manufacturer’s instructions. Serum ceruloplasmin oxidase (CPO) activity was detected by oxidation of o-dianisidine dihydrochloride (Avantor, Darmstadt, Germany), as previously described (20). This oxidation is stopped by adding sulfuric acid (9 M; Avantor) and the resulting purple-red chromophore was measured photometrically at 550 nm. The reaction was buffered in acetate buffer (0.1 M sodium acetate; Carl Roth, in 0.3% acetic acid). Serum lipopolysaccharide binding protein (LBP) was determined using ELISA (Abcam, Cat. No. ab269542), according to the manufacturer’s instructions. Liver triglycerides were determined by triglyceride colorimetric assay kit (Biomol, Hamburg, Germany). Liver content was normalized to protein levels using Pierce BCA assay (Thermo Fisher Scientific). Liver ALT activity was determined using an enzymatic assay based on ALT-catalyzed reduction of α-ketoglutarate (225 mM; Merck) to pyruvate in a reaction with L-alanine (625 mM; Carl Roth) to form L-glutamate. The reaction was buffered using Tris buffer (125 mM, pH 7.8; supplemented with L-alanine; Carl Roth, and 0.1 mM pyridoxal-5′-phosphate; Carl Roth). The subsequent conversion of pyruvate to L-lactate using lactate dehydrogenase (1.5 U/ml; Merck, Darmstadt, Germany) results in the production of NAD^+^ through the oxidation of NADH (0.23 mM; Carl Roth), which is then measured photometrically at 340 nm. Liver AST activity was measured using the AST-catalyzed reaction of α-ketoglutarate to oxaloacetate in reaction with L-aspartate (300 mM; Carl Roth). This reaction was buffered using Tris buffer (100 mM, pH 7.8; supplemented with L-aspartate and 0.1 mM pyridoxal-5′-phosphate). The subsequent reduction of oxalacetate to L-malate by Malate dehydrogenase (0.53 U/ml; Merck) led to the oxidation of NADH to NAD^+^, which was measured photometrically at 340 nm. The glutathione S-transferases (GST) activity assay is based on the GST-catalyzed reaction of 1-chloro-2,4-dinitrobenzene (CDNB, 30 mM; Th. Geyer, Renningen, Germany) in ethanol with glutathione (GSH; 100 mM; Merck) forming the conjugate GS-DNB, which is measured at 340 nm. This reaction is buffered in sodium phosphate buffer (100 mM, pH 6.5; Th. Geyer). Glutathione peroxidases (GPX) activity was measured as previously described (21), by indirect measurement of NADPH reduction in a glutathione reductase-coupled assay. NAD(P)H dehydrogenase (quinone) 1 (NQO1) activity was measured as described before (22), using 5 µl lysate and the menadion-mediated reduction of (3-(4,5-dimethylthiazol-2-yl)-2,5-diphenyltetrazolium bromide (MTT). The measurements of all enzyme activity assays were performed in triplicates by using a 96-well plate and a microplate reader (Synergy H1 or Neo2, Biotek Instruments, Bad Friedrichshall, Germany). Liver content was normalized to protein levels using Bradford analysis (Bio-Rad Laboratories, Munich, Germany).

### 2.4 Trace elements

Selenium, iron, zinc and copper concentrations were measured in mouse serum, liver lysates and in lysates of intestinal sections (ileum, colon). Samples were measured by using total reflection X-ray fluorescence spectrophotometer (TXRF, TSTAR, Bruker Nano, Berlin, Germany) as described before (23). Briefly, serum or lysates were mixed with 1 mg/l internal standard, for tissue yttrium (Merck, Cat. No. 1198090100) and for serum gallium standard (Thermo Fisher Scientific, Cat. No. 088066.AE) was added. The samples were placed on siliconized (not serum) quartz glass carriers, dried at 35–40 °C and measured for 1000 s (serum 750 s). Trace element concentrations of tissue were normalized to protein total protein concentration using Bradford assay.

### 2.5 Cytokines

Plasma and hepatic levels of C-C motif chemokine ligand 2 (CCL2), transforming growth factor-beta 1 (TGF-β1), tumor necrosis factor alpha (TNF-α), Interleukin (IL)-1α, IL-10, IL-1β, and IL-6 were measured were measured using Custom LEGENDplex Assay Panel (BioLegend, Amsterdam, Netherlands), according to the manufacturer’s instructions, with reduced sample and assay buffer volumes to 25 µl. Liver content was normalized to protein levels using BCA protein assay.

### 2.6 RT-qPCR

Liver samples were stored in RNAlater Solution (Thermo Fisher Scientific) and homogenized with RLT buffer (Qiagen, Hilden, Germany) and β-mercaptoethanol (Merck) using a rotor-stator homogenizer (TissueRuptor II, Qiagen). Total RNA was isolated from supernatant using the RNeasy Mini Kit (Qiagen) according to manufacturer’s instructions with additional on-column DNase digestion (15 min; Qiagen). cDNA was synthesized using the RevertAid First Strand cDNASynthesis Kit (Thermo Fisher Scientific). SYBR Green-based RT-qPCR (Thermo Fisher Scientific) was performed with an initial activation at 95 °C for 10 min, followed by 40 cycles of 94 °C for 15 s and 60 °C for 30 s, and a final melt curve analysis at 97 °C. Relative gene expression was calculated by the 2^−ΔΔCt^ method, normalized to the reference gene peptidylprolyl isomerase B (*Ppib)*, and expressed as log_2_(fold change (FC)) compared to the respective control group.

Intestine samples were snap frozen and total RNA was isolated with TRIzol reagent (Thermo Fisher Scientific) according to manufacturer’s instructions, followed by DNase digestion (Avantor). cDNA was synthesized using the q Script cDNASynthesis kit (Avantor). SYBR Green-based RT-qPCR (Avantor) was performed with an initial activation at 95 °C for 3 min, followed by 40 cycles of 95 °C for 15 s and 60 °C for 20 s, 72 °C for 30 s for 40 cycles, and a final melt curve analysis at 95 °C. Relative gene expression was calculated using the 2^-ΔΔCt^ method and normalized to the reference gene ribosomal protein L37 (*RPL37*). Results are shown as log_2_(FC) compared to the respective control group. The primer sequences are listed in **Supplementary Table 2**.

### 2.7 Magnetic resonance imaging and spectroscopy

All magnetic resonance imaging (MRI) and magnetic resonance spectroscopy (MRS) acquisitions were performed on a 9.4 T/20 cm bore Bio Spec scanner equipped with Avance III HD hardware and running ParaVision 6.0.1 software (Bruker, Ettlingen, Germany). Signal excitation and reception were achieved using a 2-channel mouse brain quadrature CryoProbe (Bruker). The used samples were left median lobe (LML) segments Formalin fixated for 24 h and then stored in PBS for the scans. Samples were mechanically immobilized in 15 ml plastic tubes to accommodate optimal positioning in the CryoProbe.

Hepatic fat quantification and lipid profile characterization were performed using Quantitative Magnetic Resonance Spectroscopy (MRS) with a Point-Resolved Spectroscopy (PRESS) sequence. Acquisition parameters were set as follows: echo time (TE) = 16 ms, repetition time (TR) = 5000 ms, 128 averages, and a total acquisition time (TA) of 10 min 40 s. Water suppression was achieved using Variable Power and Optimized Relaxation Delays (VAPOR), and an unsuppressed reference scan for the water signal was acquired separately with 16 averages. Spectral fitting and quantitative analysis were conducted using LCModel software (Version 6.3-1R) applying the built-in liver-8 basis set model, as described previously (24, 25). The proton density fat fraction (PDFF), was determined based on the relative signal of the 1.3 ppm lipid resonance compared to the water peak. Further lipid characterization, including the estimation of fatty acid CL and the indirect calculation of monounsaturated fatty acids (MUFA) and polyunsaturated fatty acids (PUFA) fractions, was performed according to established literature methods (26, 27), incorporating additional T_2_ decay compensation for the individual resonances.

To assess the zonal distribution of hepatic fat, a three-echo Dixon imaging approach was implemented using Magnetic Resonance Imaging (MRI). Imaging was performed using a custom-written variable flip angle Rapid Acquisition with Relaxation Enhancement (RARE) sequence (28) at a 100 µm isotropic spatial resolution. The imaging parameters were as follows: TR = 1800 ms, 2 averages, RARE factor = 50, a bandwidth = 300 kHz, and TA = 29 min (single echo). Three echoes were acquired consecutively via echo shifting to 0.417 ms, 0.179 ms, and −0.06 ms, with a nominal TE of 22.5 ms, which corresponds to the time point the central kspace line (k_0_) was acquired. The k-space encoding commenced at −0.25 using a scanner specific shifted linear wrap-around encoding scheme. The acquisition matrix was 300x150x160 (read, phase, slice) with phase encoding segmented into three shots corresponding to the RARE factor.

### 2.8 Oxylipin analysis using UPLC-MS/MS

Liver tissues were homogenized in PBS using a FastPrep-24 5G bead-beating homogenizer (M.P. Biomedicals, Irvine, CA, USA) at a speed of 6.0 m/s for 40 s using a QuickPrep adapter and 500 mg Lysing Matrix D during a single cycle without pause. The resulting homogenates were mixed with an equal volume of ice-cold LC/MS-grade methanol and kept on ice for approximately 15 min. Following centrifugation at 21,200×g for 10 min at 4 °C, a Supernatant aliquot corresponding to 25 mg of homogenized tissue was transferred into glass vials containing 1 ml PBS, 990 µl ice-cold methanol, and 10 µl of deuterium-labeled internal standard solution containing 200 nM d8-5 S-hydroxyeicosatetraenoic acid, d4-LTB4, d5-LXA4, d5-RvD2, d4-PGE2, and 10 μM d8-arachidonic acid (Cayman Chemical, Ann Arbor, MI, USA). Samples were stored at −20 °C until solid-phase extraction (SPE) and UHPLC-MS/MS analysis. Sample preparation was performed according to established protocols (29). After centrifugation at 1,200×g for 10 min at 4 °C, the supernatant was mixed with 6 ml acidified water (pH 3.5) to reduce the relative amount of organic solvent. Samples were then loaded onto pre-conditioned SPE cartridges (Sep-Pak C18–6 cc Vac Cartridge, 500 mg sorbent; Waters, Milford, MA, USA), which had been equilibrated with 6 ml methanol followed by 2 ml H_2_O. The cartridges were washed sequentially with 6 ml H_2_O and 6 ml n-hexane, and oxylipins were subsequently eluted with 6 ml methyl formate. The eluates were evaporated to dryness using a TurboVap LV solvent evaporation system (Biotage, Uppsala, Sweden) and reconstituted in 100 µl methanol/water (50:50, v/v). Oxylipin analysis was performed on a Nexera X2 UHPLC system (Shimadzu Corporation, Kyoto, Japan) coupled to a QTrap 7500 mass spectrometer (SCIEX, Marlborough, MA, USA). Chromatographic separation was achieved using an Acquity UPLC BEH C18 Column (130 Å, 1.7 µm, 2.1 mm×100 mm; Waters, Eschborn, Germany) at 50 °C with a flow rate of 0.3 ml/min. Oxylipins were identified with the help of external standards (Cayman Chemical) by confirming individual retention times and characteristic fragment ions by enhanced product ion scans (EPI). Quantification was performed using scheduled multiple reaction monitoring (sMRM) together with individual calibration curves, using the ratio of external standard peak areas to the corresponding internal standard peak areas (ES/IS). LC gradient, global MS/MS settings, oxylipin-specific parameters, including corresponding internal standards and quantification limits (LLOQs) are provided in **Supplementary Tables 3**–**5**.

### 2.9 Nomenclature

In line with the 2023 Delphi consensus statement on fatty liver disease nomenclature, this paper uses the terms “MASH” and “MASLD” as synonyms for “NASH” and “NAFLD” when discussing earlier studies (30). The nomenclature for gene and protein names follows the guidelines established by the Mouse Genome Informatics (MGI).

### 2.10 Statistics

Outliers were identified using Grubbs’ test (α = 0.05). In addition animals were excluded if they show insufficient weight gain during the experiment (< 20% after 8 weeks, < 25% after 12 weeks, or < 30% after 16 or 24 weeks from baseline weight) or if liver abnormalities were present (e.g. individual values, with whiskers extending from the minimum to the maximum and the median indicated by a horizontal line. For longitudinal measurements (body weight gain), individual data points were connected by lines to illustrate temporal changes. Mean ± standard deviation (SD) are shown. Statistical comparisons between groups were performed using one-way ANOVA followed by Holm-Sidak’s multiple comparisons *post-hoc* test. *p* < 0.05 was considered statistically significant. In heatmaps P-values were corrected using two-stage linear step-up procedure of Benjamini, Krieger, and Yekutieli. Bold text indicates *p* < 0.05 vs. control.

## 3 Results

### 3.1 *Apoe*^-/-^ mice demonstrate accelerated MASH development compared to wild-type mice

**Figure 1:**
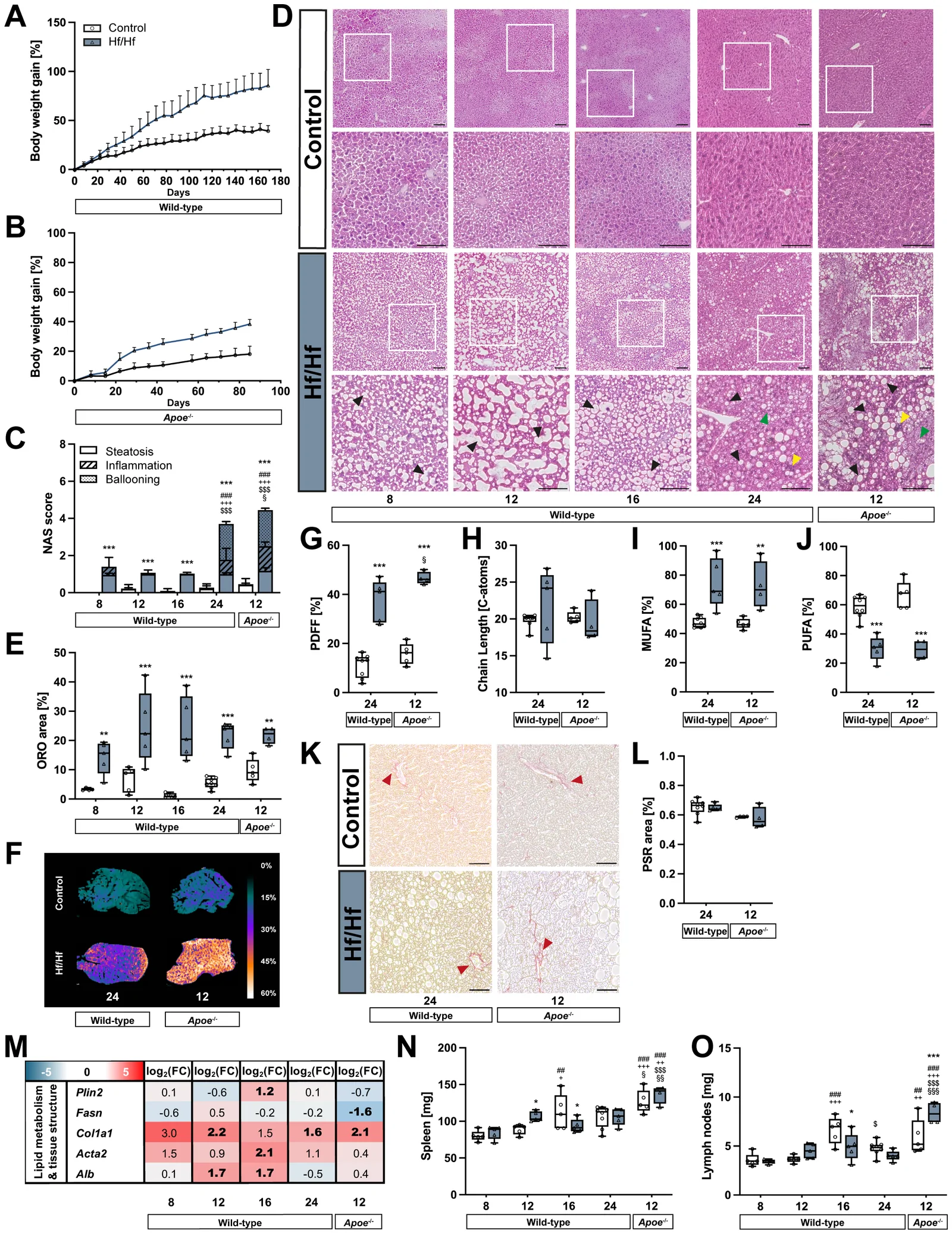
Influence of high-fat/high-fructose (Hf/Hf) diet on MASH development in wild-type and *Apoe*^-/-^ mice. Duration of diet 8–24 weeks in wild-type and 12 weeks in *Apoe*^-/-^. **(A)** Body weight gain in wild-type and **(B)** *Apoe*^-/-^ mice. **(C)** Nonalcoholic fatty liver disease activity score (NAS). **(D)** Representative hematoxylin and eosin (H&E) images of liver sections. Arrows indicate fat vacuoles of different sizes (black), inflammatory cell infiltration (yellow), hepatocellular ballooning (green). Overview images are shown with corresponding higher magnifications below (scale bars are 100 µm). **(E)** Quantitative analysis of Oil Red O (ORO)-positive area. **(F)** Representative magnetic resonance imaging (MRI)-derived liver fat maps, and quantification of **(G)** proton density fat fraction (PDFF), **(H)** fatty acid chain length, **(I)** percentage of monounsaturated fatty acids (MUFA), and **(J)** polyunsaturated fatty acids (PUFA) by using quantitative magnetic resonance spectroscopy (MRS) of liver samples. **(K)** Representative Picro Sirius Red (PSR) images of liver sections. Red arrows indicate PSR-positive (fibrotic) areas. Scale bars are 100 µm. **(L)** Quantitative analysis of PSR-positive areas. **(M)** Heatmap of hepatic mRNA expression of various genes. Shown as log_2_ of fold change (FC) compared to the respective control. **(N)** Weight of spleen, and **(O)** axillary lymph nodes. **(A–C)** n = 4–8. Data are presented as mean ± SD. **(E, G–J, L, N, O)** Boxes represent the interquartile range (25th–75th percentiles), with the median as the center line. Whiskers extend from the minimum to the maximum value. Individual data points are shown. Sample sizes varied between analyses due to assay-specific sample availability and exclusion of statistical outliers as described in the statistical analysis section. **(C–L, N, O)** Statistical analysis was performed using two-way ANOVA followed by Holm-Sidak *post-hoc* test. *p < 0.05, **p < 0.01, ***p < 0.001 vs. control; # vs. 8 weeks, + vs. 12 weeks, $ vs. 16 weeks, § vs. 24 weeks; **(M)** n = 4–8. Statistical analysis was performed using one-way ANOVA based on ΔCt-values followed by Holm-Sidak *post-hoc* test. P-values were corrected using two-stage linear step-up procedure of Benjamini, Krieger, and Yekutieli. Bold text indicates *p* < 0.05 vs. control.

The anticipated outcome was achieved, as Hf/Hf feeding led to an increased body weight (BW) gain at all time points in both genotypes compared to controls (**Figures 1A, B**). After 12 weeks, BW gain was 29% higher in wild-type and 20% higher in *Apoe*^-/-^ mice fed Hf/Hf diet compared to controls (*p* < 0.001). After 24 weeks, BW gain in wild-type mice further increased to 41% compared to controls (p < 0.001). Notably, BW development showed lower interindividual variability in *Apoe*^-/-^ compared to wild-type mice (coefficient of variation (CV) in the Hf/Hf group: 8.0% in *Apoe*^-/-^ vs. 20.5% averaged across all time points in wild-type; **Figures 1A, B**).

Histological assessment using the NAS was performed to evaluate MASLD-related parameters in H&E-stained liver sections. *Apoe*^-/-^ mice exhibited significantly higher total NAS values than wild-type mice at all analyzed times (4.5 in *Apoe*^-/-^ mice at 12 weeks vs. 1.4, 1.1, 1.0, and 3.7 in wild-type mice at 8, 12, 16, and 24 weeks, respectively; *p* < 0.001 vs. control; **Figure 1C**). This difference was driven by consistently elevated scores across all NAS components, with inflammation showing the most pronounced increase. *Apoe*^-/-^ mice reached inflammation scores of 1–2 as early as 12 weeks, whereas wild-type mice rarely exceeded a score of 1 even after 24 weeks of Hf/Hf feeding (**Figure 1C**). Accordingly, H&E-stained liver sections of wild-type mice after 24 weeks and *Apoe*^-/-^ mice after 12 weeks of Hf/Hf feeding revealed pronounced steatotic alterations, characterized by steatosis (black arrows), hepatocellular ballooning (green arrows), and lobular inflammatory infiltrates (yellow arrows) (**Figure 1D**). Oil Red O (ORO) staining demonstrated pronounced hepatic lipid accumulation in both genotypes following Hf/Hf diet. Quantitative analysis of ORO-positive areas revealed comparable levels of neutral lipid accumulation in wild-type and *Apoe*^-/-^ mice feed a Hf/Hf diet at all times (16%; *p* < 0.01, 22%; *p* < 0.001, 20%; *p* < 0.001, and 24%; *p* < 0.001 vs. control in wild-type mice at 8, 12, 16, and 24 weeks, respectively and 22%; *p* < 0.01 vs. control in *Apoe*^-/-^ mice at 12 weeks), with steatosis reaching a plateau after 12 weeks of dietary intervention (**Figure 1E**). To further characterize hepatic lipid composition, MRI and MRS were performed. These analyses revealed differences between wild-type mice after 24 weeks of Hf/Hf feeding and *Apoe*^-/-^ mice after 12 weeks (**Figure 1F**). This was supported by quantitative MRS, showing a higher PDFF, indicating increased hepatic fat accumulation, in *Apoe*^-/-^ mice compared to wild-type mice (46% vs. 41%, respectively; *p* < 0.05; **Figure 1G**). Again, *Apoe*^-/-^ mice showed lower interindividual variability in compared to wild-type (CV in the Hf/Hf group: 5.5% in *Apoe*^-/-^ vs. 22.8% in wild-type; **Figure 1G**). While no differences in fatty acid chain length were detected between genotypes, increased variability was observed in both Hf/Hf-fed groups (**Figure 1H**). The relative proportion of MUFA increased from 50% to 70% after Hf/Hf diet in both genotypes (wild-type: *p* < 0.001; *Apoe*^-/-^: *p* < 0.01 vs. control; **Figure 1I**), whereas PUFA were reduced from 60–70% to 30% following Hf/Hf feeding in both genotypes (*p* < 0.001 vs. control; **Figure 1J**). Alterations in hepatic lipid metabolism were further assessed by mRNA expression analyses of relevant genes (**Figure 1M**). Perilipin 2 (*Plin2*) expression, possibly indicating increased lipid storage, was strongly upregulated in wild-type mice after 16 weeks of Hf/Hf feeding (FC = 1.2; *p* < 0.05). In contrast, fatty acid synthase (*Fasn*) expression was downregulated in *Apoe*^-/-^ mice after 12 weeks of Hf/Hf feeding (FC = −1.6; *p* < 0.05; **Figure 1M**), which may be an indicator for reduced *de novo* lipogenesis.

PSR staining revealed no evidence of advanced fibrosis in either genotype at the latest time point (**Figures 1K, L**). Collagen deposition was primarily localized to perisinusoidal areas in both genotypes (**Figure 1K**, red arrow). To further assess fibrotic remodeling, hepatic mRNA expression of fibrosis-associated genes was analyzed by RT-qPCR (**Figure 1M**). mRNA expression of collagen type I alpha 1 (*Col1a1*) and actin alpha 2 (*Acta2*) was upregulated in both genotypes across all time points (log_2_(FC) range = 1.5–3.0 and 0.4–2.1, respectively). Albumin (*Alb*) expression was increased in wild-type mice after 12 and 16 weeks of Hf/Hf feeding (log_2_(FC) = 1.7; *p* < 0.01, both) but declined at 24 weeks (log_2_(FC) = −0.5). In *Apoe*^-/-^ mice, *Alb* showed a mild upregulation after 12 weeks of Hf/Hf feeding (log_2_(FC) = 0.4; **Figure 1**).

Changes in secondary lymphoid organs were assessed by spleen and axillary lymph node weights following Hf/Hf feeding (**Figures 1N, O**). In wild-type mice, spleen weight was not significantly altered after 8 weeks of Hf/Hf diet compared to controls (79.2 mg vs. 89.0 mg). After 12 weeks, a substantial rise was observed, with spleen weight increasing from 92.5 mg to 102.7 mg (*p* < 0.05), followed by a reduction at 16 weeks (109.4 mg vs. 91.0 mg; *p* < 0.05). At this time point, spleen weights showed marked interindividual variability, particularly in the control group (CV: 21.5%; **Figure 1M**). After 24 weeks of Hf/Hf feeding, spleen weight in wild-type mice was no longer significantly different from controls (112.9 mg vs. 107.0 mg). *Apoe*^-/-^ mice did not exhibit a significant increase in spleen weight at the investigated time point following Hf/Hf feeding (122.0 mg vs. 141.2 mg). Nevertheless, in the control feeding group, *Apoe*^-/-^ mice exhibited significantly higher absolute spleen weights than wild-type mice at all time points analyzed (92.5 mg vs. 122.0 mg, respectively after control diet for 12 weeks; *p* < 0.001), indicating a genotype-dependent difference in baseline spleen mass (**Figure 1N**). Axillary lymph nodes were significantly enlarged in *Apoe*^-/-^ mice from 5.2 mg to 8.3 mg (*p* < 0.001) after 12 weeks of Hf/Hf feeding. In wild-type mice, lymph node weight showed a modest yet significant decrease after 16 weeks of Hf/Hf feeding compared to control (*p* < 0.05), with no differences detected at other time points (**Figure 1O**). Together, these data demonstrate genotype-specific differences in lymphoid organ involvement in response to Hf/Hf diet, with *Apoe*^-/-^ mice showing increased lymphoid tissue mass despite the absence of diet-induced splenomegaly.

**Figure 2:**
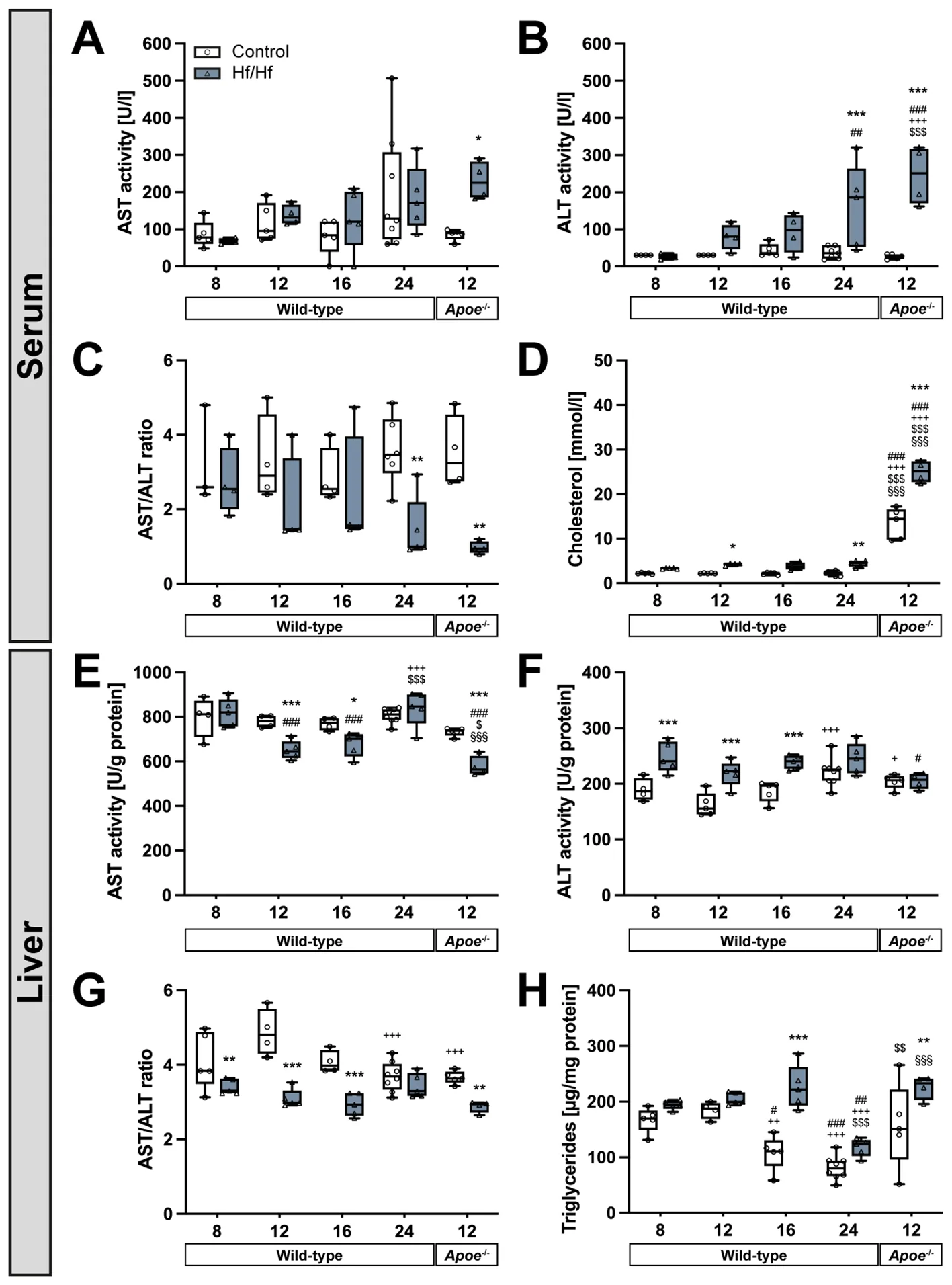
Changes of serum and hepatic markers for liver function and lipid metabolism after high-fat/high-fructose (Hf/Hf) diet. Duration of diet 24 weeks in wild-type and 12 weeks in *Apoe*^-/-^. **(A)** Serum aspartate aminotransferase (AST) activity, **(B)** alanine aminotransferase (ALT) activity, **(C)** AST-ALT ratio, and **(D)** total cholesterol. **(E)** Hepatic AST activity, **(F)** ALT activity, **(G)** AST-ALT ratio, and **(H)** triglyceride content in liver tissue are shown. Boxes represent the interquartile range (25th–75th percentiles), with the median as the center line. Whiskers extend from the minimum to the maximum value. Individual data points are shown. Sample sizes varied between analyses due to assay-specific sample availability and exclusion of statistical outliers as described in the statistical analysis section. Statistical analysis was performed using two-way ANOVA followed by Holm-Sidak *post-hoc* test. *p < 0.05, **p < 0.01, ***p < 0.001 vs. control; # vs. 8 weeks, + vs. 12 weeks, $ vs. 16 weeks, § vs. 24 weeks.

Serum liver enzyme activity exhibited a time- and genotype-dependent response to the Hf/Hf diet (**Figures 2A–C**). Serum AST levels changed by -12%, +38%, +43%, and +33% vs. control after 8, 12, 16, and 24 weeks of Hf/Hf feeding in wild-type mice. Whereas *Apoe*^-/-^ mice showed a marked increase by 150% vs. control (*p* < 0.05) already after 12 weeks. Serum ALT activity showed a time-dependent response to Hf/Hf feeding in wild-type mice, with a slightly initial decrease of 10% vs. control at 8 weeks followed by marked increases at later time points by 170% after 12 weeks and 175% after 16 weeks. After 24 weeks of Hf/Hf diet an increase by 417% (*p* < 0.001 vs. control) could be observed. In contrast, *Apoe*^-/-^ mice exhibited a markedly greater elevation, with ALT levels already increased by 828% after 12 weeks (*p* < 0.001 vs. control). The serum AST/ALT ratio declined over time and in *Apoe*^-/-^ mice, reflecting a stronger increase of ALT. The serum AST/ALT ratio was reduced in wild-type mice only after 24 weeks of Hf/Hf diet (−71%; *p* < 0.01 vs. control), whereas the same reduction was observed in *Apoe*^-/-^ mice already after 12 weeks (*p* < 0.01 vs. control; **Figure 2C**). Total serum cholesterol levels were significantly higher in *Apoe*^-/-^ control mice compared with wild-type controls (2.2 mmol/l vs. 14.4 mmol/l, in wild-type at 12 weeks and *Apoe*^-/-^, respectively; *p* < 0.001) and were further elevated after 12 weeks of Hf/Hf feeding (4.4 mmol/l, *p* < 0.05 and 25.1 mmol/l, *p* < 0.001 vs. control; in wild-type and *Apoe*^-/-^, respectively; **Figure 2D**).

Analysis of hepatic enzyme activities (**Figures 2E–G**), show reduced AST activity in wild-type mice after 12 weeks (−17%; *p* < 0.001 vs. control) and after 16 weeks of Hf/Hf diet (−9%; *p* < 0.05 vs. control) as well as in *Apoe*^-/-^ mice (−24%; *p* < 0.001 vs. control) (**Figure 2E**). Hepatic ALT activity was increased in wild-type after Hf/Hf feeding (+29%, +43%, and +22% in wild-type mice at 8, 12, and 16 weeks, respectively; *p* < 0.001 vs. control) and no significant differences after 24 weeks in wild-type and *Apoe*^-/-^ mice (**Figure 2F**). The hepatic AST/ALT ratio decreased over time in both genotypes, indicating progressive liver damage (−14%, −38%, −26%, and −11%, in wild-type mice at 8, 12, 16, and 24 weeks, respectively and −19%, in *Apoe*^-/-^ mice; **Figure 2G**) upon Hf/Hf feeding. Hepatic triglyceride content (**Figure 2H**) was markedly elevated after 16 weeks of Hf/Hf feeding in wild-type mice (222 µg/mg protein, *p* < 0.001 vs. control) and after 12 weeks in *Apoe*^-/-^ (233 µg/mg protein, *p* < 0.01 vs. control), confirming steatotic progression.

Because wild-type mice at 24 weeks and *Apoe*^-/-^ mice at 12 weeks exhibited the most advanced MASH phenotype, reflected by markedly increased NAS scores (3.7 ± 0.7; *p* < 0.001 and 4.5 ± 0.2; *p* < 0.001, respectively) and a reduced serum AST/ALT ratio (both −71%; *p* < 0.01 vs. control), subsequent analyses were restricted to these two groups. Earlier times in wild-type mice (8, 12, and 16 weeks) did not show comparable disease severity and were therefore not included in subsequent investigations.

### 3.2 Enhanced and accelerated inflammatory response in *Apoe*^-/-^ mice

**Figure 3:**
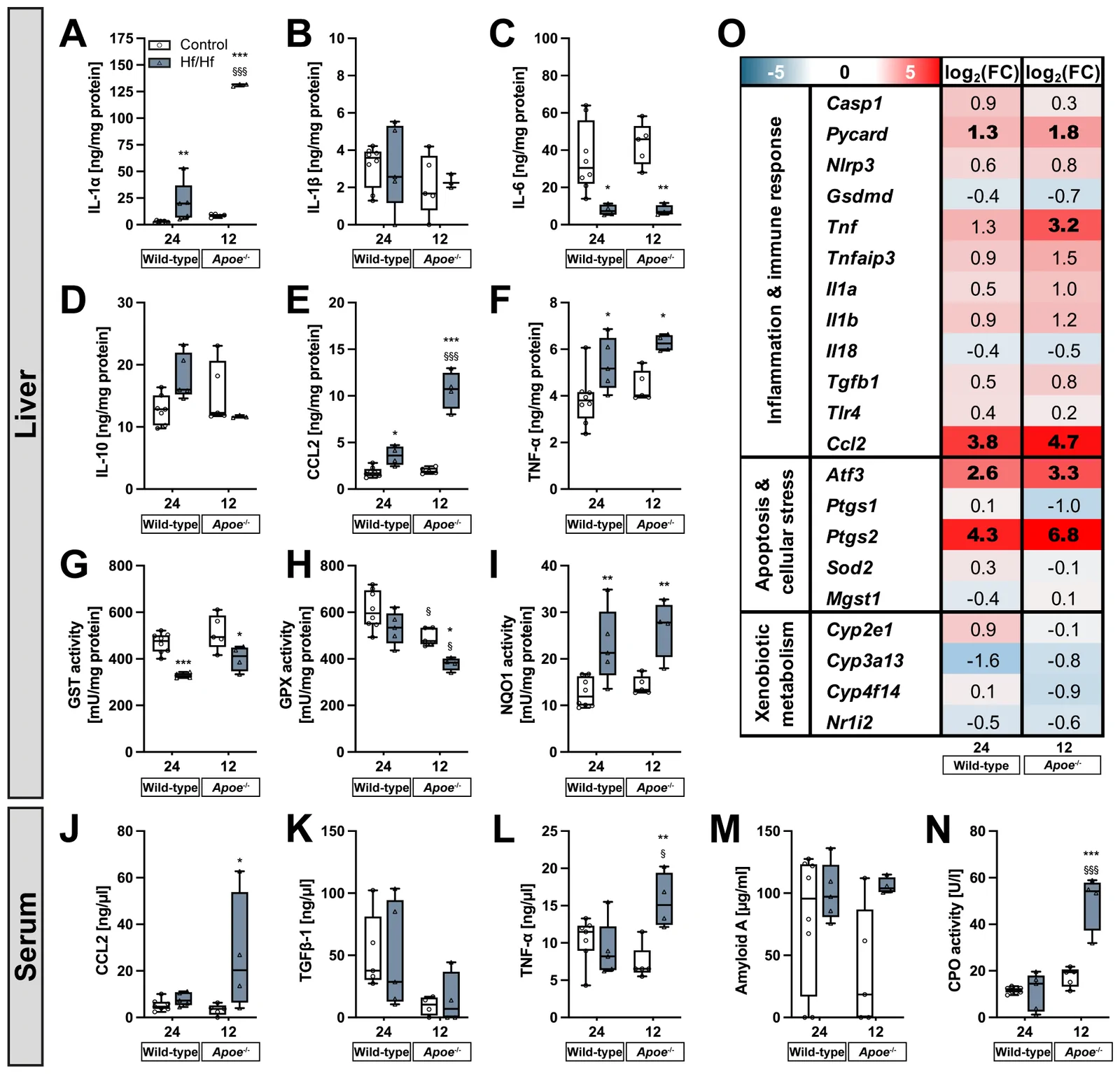
Inflammatory markers in liver and serum after high-fat/high-fructose (Hf/Hf) diet. Duration of diet 24 weeks in wild-type and 12 weeks in *Apoe*^-/-^. **(A)** Hepatic levels of interleukin (IL)-1α, **(B)** IL-1β, **(C)** IL-6, **(D)** IL-10, **(E)** C-C motif chemokine ligand 2 (CCL2), **(F)** tumor necrosis factor-α (TNF-α), **(G)** glutathione S-transferase (GST) activity, **(H)** glutathione peroxidase (GPX) activity, and **(I)** NAD(P)H dehydrogenase (quinone) 1 (NQO1) activity. Serum cytokines including **(J)** CCL2, **(K)** transforming growth factor-β1 (TGF-β1), **(L)** TNF-α, **(M)** serum amyloid A, and **(N)** ceruloplasmin oxidase (CPO) activity are shown. **(O)** Heatmap of hepatic mRNA expression of various inflammatory genes. Shown as log_2_(fold change (FC)) compared to the respective control. **(A–N)** Boxes represent the interquartile range (25th–75th percentiles), with the median as the center line. Whiskers extend from the minimum to the maximum value. Individual data points are shown. Sample sizes varied between analyses due to assay-specific sample availability and exclusion of statistical outliers as described in the statistical analysis section. Statistical analysis was performed using two-way ANOVA followed by Holm-Sidak *post-hoc* test. *p < 0.05, **p < 0.01, ***p < 0.001 vs. control; § vs. 24 weeks. **(O)** n = 4–8. Statistical analysis was performed using one-way ANOVA based on ΔCt-values followed by Holm-Sidak *post-hoc* test. P-values were corrected using two-stage linear step-up procedure of Benjamini, Krieger, and Yekutieli. Bold text indicates *p* < 0.05 vs. control.

In liver lysates of Hf/Hf-fed mice, *Apoe*^-/-^ mice exhibited a markedly stronger inflammatory response compared with wild-type mice, as reflected by significantly increased levels of interleukin 1 alpha (IL-1α) (+1364% vs. +665%; p < 0.001, respectively; **Figure 3A**), CCL2 (+472% vs. +112%; p < 0.001; **Figure 3E**), and TNF-α (+55% vs. +36%; **Figure 3F**). In line with diet-induced hepatic stress, cellular stress markers were further assessed. The activities of GST and GPX were significantly reduced in *Apoe*^-/-^ mice following Hf/Hf feeding (−17% and −20%, respectively; p < 0.05 vs. control; **Figures 3G, H**), indicating an impaired antioxidant defense. In contrast, hepatic NQO1 activity was markedly increased (+108%; p < 0.01 vs. control; **Figure 3I**), consistent with a compensatory activation of stress-responsive pathways. In wild-type mice, Hf/Hf feeding resulted in a comparable reduction in GST and GPX activities (−31%; p < 0.001 vs. control; and −10%, respectively; **Figures 3G, H**), and increase of NQO1 activity (+79%, p < 0.01 vs. control; **Figure 3I**) as in *Apoe*^-/-^ mice.

In serum of *Apoe*^-/-^ mice increased levels of CCL2 (+451%; *p* < 0.05; **Figure 3J**), TNF-α (+132%; *p* < 0.01; **Figure 3L**), serum amyloid A (SAA) (+462%; **Figure 3M**) and CPO (+179%; *p* < 0.001 vs. control; **Figure 3N**) were observed, whereas serum levels of wild-type mice remained unchanged (**Figures 3J–N**). RT-qPCR analysis of liver tissue revealed differential expression patterns of key genes between the two mouse genotypes, involving inflammation and immune response as well as apoptosis and cellular stress. *Apoe*^-/-^ mice showed enhanced expression of pro-inflammatory genes, including cytokines, chemokines, and inflammatory cell markers, consistent with the findings of increased inflammation compared to wild-type mice (**Figure 3O**).

**Figure 4:**
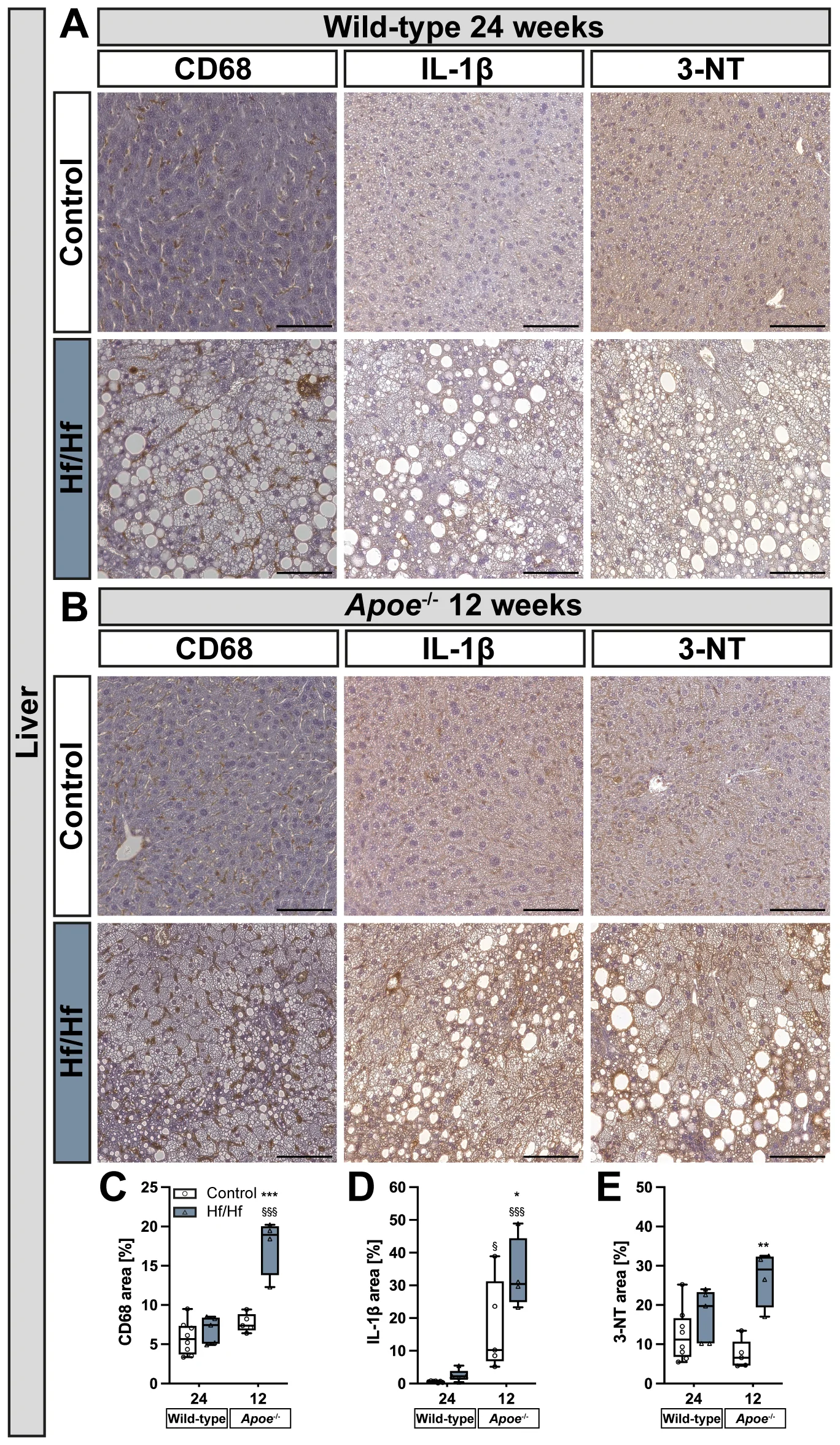
Immunohistochemical analysis of liver inflammation and oxidative stress markers after high-fat/high-fructose (Hf/Hf) diet. Duration of diet 24 weeks in wild-type and 12 weeks in *Apoe*^-/-^. Representative images of immunohistochemistry (IHC) staining of cluster of differentiation 68 (CD68), interleukin-1β (IL-1β), and 3-nitrotyrosine (3-NT) in liver sections from **(A)** C57BL/6 mice and **(B)** *Apoe*^-/-^ mice. Scale bars are 100 µm. Quantitative analysis of **(C)** CD68, **(D)** IL-1β, and **(E)** 3-NT is shown. Boxes represent the interquartile range (25th–75th percentiles), with the median as the center line. Whiskers extend from the minimum to the maximum value. Individual data points are shown. Sample sizes varied between analyses due to assay-specific sample availability and exclusion of statistical outliers as described in the statistical analysis section. **(C–E)** Statistical analysis was performed using two-way ANOVA followed by Holm-Sidak *post-hoc* test. *p < 0.05, **p < 0.01, ***p < 0.001 vs. control; § vs. 24 weeks.

To evaluate macrophage infiltration and its associated inflammatory and oxidative responses, hepatic expression of CD68, IL-1β, and 3-NT was analyzed. Analysis of CD68 demonstrated pronounced genotype-dependent differences in macrophage infiltration. *Apoe*^-/-^ mice displayed extensive accumulation of CD68+ cells in the liver after 12 weeks of Hf/Hf diet feeding compared with wild-type mice after 24 weeks of Hf/Hf diet feeding (19% vs. 8%, respectively; *p* < 0.001; **Figures 4A–C**), indicating enhanced macrophage recruitment associated. As macrophages represent a major cellular source of the pro-inflammatory cytokine IL-1β, its expression was subsequently examined. Both the cellular localization and staining intensity of IL-1β were markedly increased in *Apoe*^-/-^ control mice compared with wild-type controls (10% vs. 1%; respectively; *p* < 0.05; **Figures 4A, B, D**). A further increase was observed in mice fed a Hf/Hf diet, with 30% of IL-1β positive area in *Apoe*^-/-^ and 2% in wild-type mice (*p* < 0.001), suggesting an additive effect in *Apoe*^-/-^ of dietary challenge on hepatic inflammation (**Figures 4A, B, D**). In addition, hepatic levels of 3-NT, a marker of oxidative and nitrative stress, were significantly elevated in Hf/Hf diet-fed *Apoe*^-/-^ (29%; *p* < 0.01 vs. control; **Figures 4A, B, E**). Increased oxidative stress may contribute to the exacerbated hepatocellular injury and inflammatory responses observed in the *Apoe*^-/-^ model.

### 3.3 Oxylipin and lipid mediator profiles differ between genotypes

**Figure 5:**
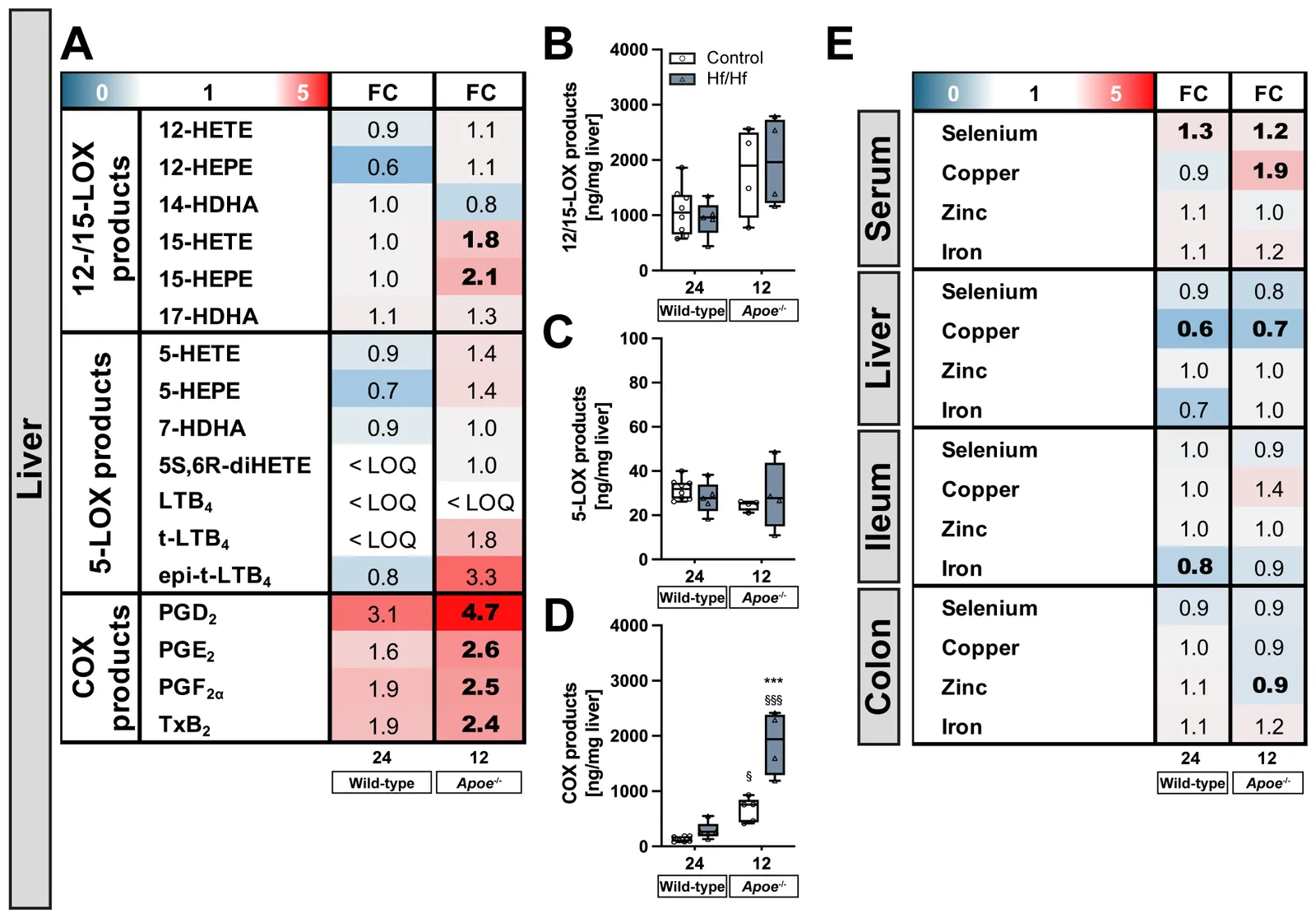
Lipidomic analysis and changes of trace element concentrations in serum and tissues serum after high-fat/high-fructose (Hf/Hf) diet. Duration of diet 24 weeks in wild-type and 12 weeks in *Apoe*^-/-^. **(A)** Heatmap showing fold change (FC) of hepatic oxylipin species compared to the respective control. If the oxylipin concentrations in a group were below and quantification limit (LLOQ), 1/2 LLOQ was used for the corresponding oxylipin. Quantitative levels of **(B)** 12/15-lipoxygenase (12/15-LOX) products, **(C)** 5-lipoxygenase (5-LOX), and **(D)** cyclooxygenase (COX). **(E)** Heatmap of trace element levels in serum, liver, ileum, and colon are shown as fold change relative to control. **(B–D)** Boxes represent the interquartile range (25th–75th percentiles), with the median as the center line. Whiskers extend from the minimum to the maximum value. Individual data points are shown. Sample sizes varied between analyses due to assay-specific sample availability and exclusion of statistical outliers as described in the statistical analysis section. Statistical analysis was performed using two-way ANOVA followed by Holm-Sidak *post-hoc* test. *p < 0.05, **p < 0.01, ***p < 0.001 vs. control; § vs. 24 weeks; **(A, E)** n = 4–8. Statistical analysis was performed using one-way ANOVA based on absolute concentrations followed by Holm-Sidak *post-hoc* test. P-values were corrected using two-stage linear step-up procedure of Benjamini, Krieger, and Yekutieli. Bold text indicates *p* < 0.05 vs. control.

UPLC-MS/MS analysis of oxylipins in the liver revealed distinct lipid mediator profiles between mouse genotypes. *Apoe*^-/-^ mice showed even more elevated levels of immunomodulatory oxylipins derived from arachidonic acid, including cyclooxygenase (COX)-derived prostaglandins (PG) (esp. PGD_2_; FC = 4.7) and pro-inflammatory 5-lipoxygenase (LOX)-derived leukotrienes (esp. epi-t-LTB_4_; FC = 3.3), consistent with an enhanced inflammatory response (**Figures 5A, D**). The overall quantity of 12/15-LOX-products was higher in *Apoe*^-/-^ than wild-type mice, independent of the dietary intervention (1964 ng/mg vs. 961 ng/mg, respectively after Hf/Hf diet; *p* < 0.05; **Figure 5B**). This dysregulated oxylipin profile likely contributes to the sustained inflammatory phenotype observed in *Apoe*^-/-^ mice.

### 3.4 Hf/Hf diet alters trace element homeostasis

The analysis of trace element concentrations in liver, serum, and intestinal tissues revealed Hf/Hf diet dependent changes in trace element homeostasis (**Figure 5E**). Copper was particularly affected, with decreased concentrations in the liver in both genotypes (FC = 0.6; *p* < 0.01 vs. control in wild-type and 0.7; *p* < 0.01 vs. control; in *Apoe*^-/-^) and increased concentrations in serum and ileum in *Apoe*^-/-^ mice (FC = 1.9; *p* < 0.001 and 1.4; respectively). Serum concentrations of selenium, which is important for antioxidant defense as part of selenoproteins, were elevated after Hf/Hf feeding in wild-type mice (FC = 1.3; *p* < 0.01) and *Apoe*^-/-^ mice (FC = 1.2; *p* < 0.01). In the liver, colon, and ileum, however, selenium concentrations were slightly reduced in both genotypes. The intestinal distribution of iron also differed. Wild-type mice showed lower concentrations in the ileum (FC = 0.8; *p* < 0.01), whereas *Apoe*^-/-^ mice had higher concentrations in the colon (FC = 1.2). These differences may contribute to variations in oxidative stress between the two genotypes. Zinc concentrations were largely stable in both genotypes after Hf/Hf diet. However, in the colon, *Apoe*^-/-^ mice exhibited a modest but statistically significant reduction in zinc levels after Hf/Hf diet (FC = 0.9; *p* < 0.001).

### 3.5 Intestinal barrier function and gut–liver axis

**Figure 6:**
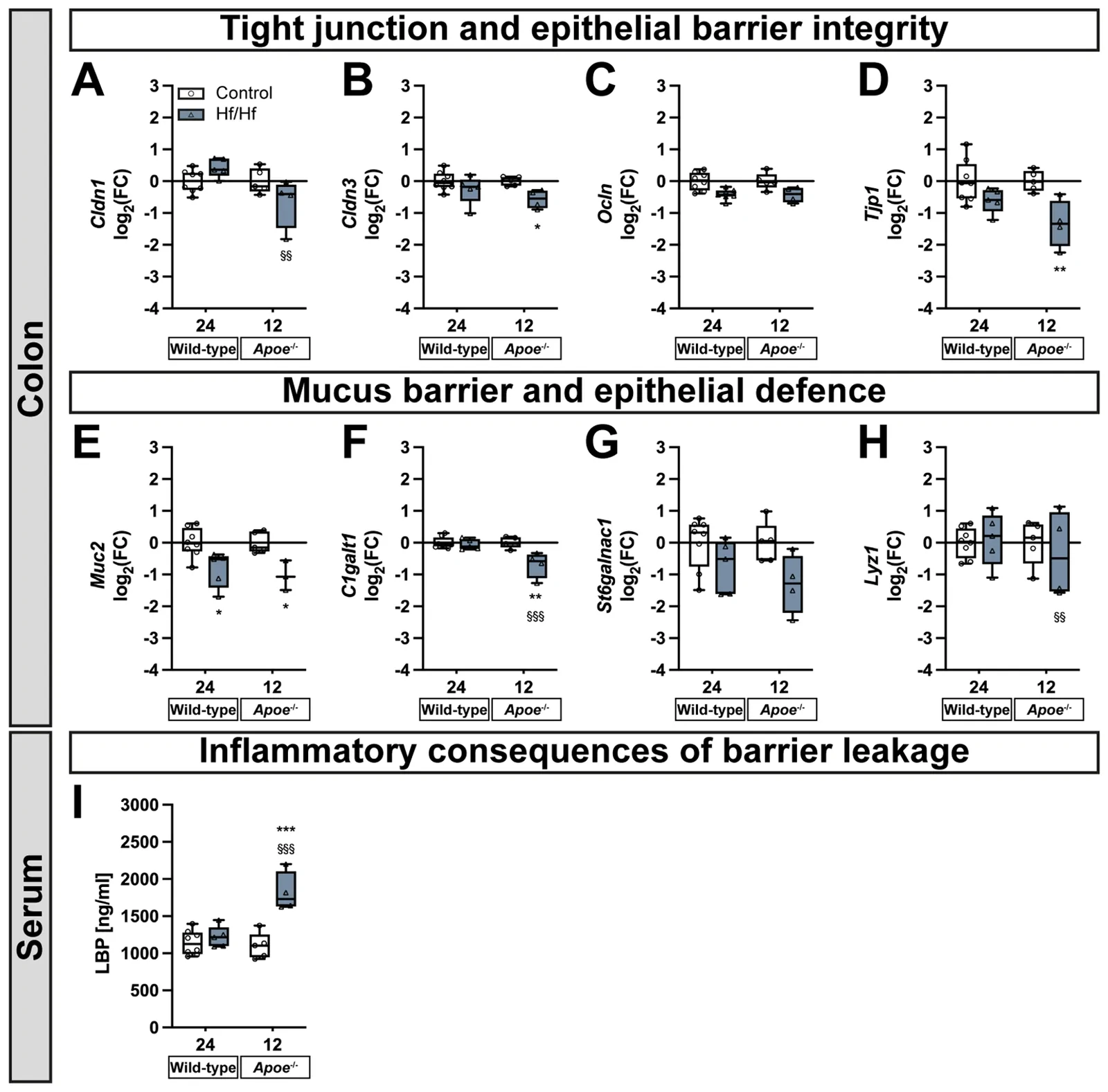
Analysis of markers of intestinal barrier integrity after high-fat/high-fructose (Hf/Hf) diet. Duration of diet 24 weeks in wild-type and 12 weeks in *Apoe*^-/-^. Relative mRNA levels were measured in colon for **(A)** claudin 1 (*Cldn1*), **(B)** claudin 3 (*Cldn3*), **(C)** occludin *(Ocln)*, **(D)** tight junction protein 1 (*Tjp1*), **(E)** mucin 2 (*Muc2*), **(F)** core 1 synthase, glycoprotein-N-acetylgalactosamine 3-beta-galactosyltransferase, 1 (*C1galt1*), **(G)** ST6 (alpha-N-acetyl-neuraminyl-2,3-beta-galactosyl-1,3)-N-acetylgalactosaminide alpha-2,6-sialyltransferase 1 ( *St6galnac1*), and **(H)** lysozyme 1 (*Lyz1*). **(I)** Serum concentrations of lipopolysaccharide-binding protein (LBP). **(A–H)** Data are displayed as log_2_ (fold change (FC)) relative to control. **(A–I)** Boxes represent the interquartile range (25th–75th percentiles), with the median indicated as center line. Whiskers extend from the minimum to the maximum value. Individual data points are shown. Sample sizes varied between analyses due to assay-specific sample availability and exclusion of statistical outliers as described in the statistical analysis section. Statistical analysis was performed using two-way ANOVA followed by Holm-Sidak *post-hoc* test. *p < 0.05, **p < 0.01, ***p < 0.001 vs. control; § vs. 24 weeks.

Gene expression of several intestinal barrier markers was analyzed in the ileum and colon after 12 and 24 weeks of Hf/Hf feeding in *Apoe*^-/-^ and wild-type mice, respectively. Changes were mostly limited to the colon (**Figures 6A–D**), while the expression levels in the ileum remained unchanged (**Supplementary Figure 1**). In the colon, Hf/Hf diet reduced expression of tight junction genes such as claudin *(Cldn) 3* (log_2_ (FC) = −0.2 in wild-type and −0.5 in *Apoe*^-/-^; *p* < 0.05 vs. control), occludin (*Ocln*) (log_2_ (FC) = −0.4 in both), and tight junction protein 1 (*Tjp1*) (log_2_ (FC) = −0.6 in wild-type and −1.3 in *Apoe*^-/-^; *p* < 0.01 vs. control), as well as the mucus-related genes mucin 2 (*Muc2*) (log_2_ (FC) = −0.5 in wild-type; *p* < 0.05 vs. control, and −1.1 in *Apoe*^-/-^; *p* < 0.05 vs. control), and ST6 (alpha-N-acetyl-neuraminyl-2,3-beta-galactosyl-1,3)-N-acetylgalactosaminide alpha-2,6-sialyltransferase 1 (*St6galnac1*) (log_2_ (FC) = −0.5 in wild-type and −1.3 in *Apoe*^-/-^), (**Figures 6B–G**). These alterations indicate a region-specific susceptibility of the colonic barrier to metabolic dietary stress. *Apoe*^-/-^ mice displayed an additional reduction in *Cldn1* (log_2_ (FC) = −0.4; **Figure 6A**) and core 1 synthase, glycoprotein-N-acetylgalactosamine 3-beta-galactosyltransferase, 1 (*C1galt1*) (log_2_ (FC) = −0.6; *p* < 0.01 vs. control; **Figure 6F**) in comparison to *Apoe*^-/-^ control. As well as a reduced expression of lysozyme 1 *(Lyz1)* in comparison to the Hf/Hf treated group wild-type mice (*p* < 0.01; **Figure 6H**).

The functional relevance of these molecular alterations was reflected systemic. Only in *Apoe*^-/-^ mice Hf/Hf diet increased serum lipopolysaccharide-binding protein (LBP) levels significant to 1730 ng/ml (+57%; *p* < 0.001; **Figure 6I**). These data suggest that APOE deficiency aggravates diet-induced intestinal barrier dysfunction and promotes translocation of microbial products to the liver, thereby contributing to activation of the gut–liver axis and progression of hepatic inflammation. Together, these results show that Hf/Hf diet causes region- and genotype-specific alterations of the intestinal barrier, with the distal colon as the main site of dysfunction. *Apoe*^-/-^ mice display a more pronounced and functionally relevant impairment, linking intestinal barrier failure to hepatic inflammation and metabolic disease progression.

## 4 Discussion

### 4.1 Temporal progression of MASH in wild-type mice: from steatosis to inflammation

The presented longitudinal study of wild-type mice fed an Hf/Hf diet shows a clear temporal gap between steatosis and inflammation. Steatosis emerges early on and remains stable between 12 and 24 weeks. However, significant hepatic inflammation only manifests after 24 weeks. This temporal pattern aligns with the established “multiple-hit” paradigm of MASH and MALSD pathogenesis, wherein initial lipid accumulation (“first hit”) and other “hits”, including oxidative stress, mitochondrial dysfunction, and inflammation, can lead to progressive liver damage and MASH (4). Previous studies in wild-type mice have documented similar outcomes, with steatosis appearing within 8–9 weeks of high-fat feeding and steatohepatitis emerging between 16–28 weeks (6, 31–33). The here presented findings add to these observations by showing that after 16 weeks, despite ongoing fat build-up, the inflammatory reaction is mostly absent in wild-type wild-type mice.

The inflammatory response and hepatocellular damage observed at 24 weeks in wild-type was characterized by increased NAS scores, elevated hepatic pro-inflammatory cytokines (IL-1α, CCL2, and TNF-α), and reduced serum AST/ALT ratio. This constellation of markers indicates a beginning progression from steatosis to steatohepatitis. The elevation of hepatic IL-1α and TNF-α suggests inflammasome activation and macrophage polarization toward a pro-inflammatory phenotype (34, 35). Interestingly, a paradoxical reduction in hepatic IL-6 at this time was observed; however, hepatic IL-6 mRNA expression could not be assessed due to undetectable transcript levels. Such a decline may indicate a dysregulated regenerative response or an adaptive change in the liver under sustained oxidative stress and inflammation. Although IL-6 is generally considered to be a pro-inflammatory cytokine, it is important to distinguish between the classical/*cis* IL-6 signaling via membrane-bound IL-6-receptor (IL-6R), which activates the hepatoprotective gp130-signal transducer and activator of transcription 3 (STAT3) signaling pathway, and supports regeneration, mitochondrial function, and metabolic homeostasis (36, 37) and the IL-6 *trans* signaling, which, via soluble IL-6R, leads to enhanced pro-inflammatory processes that are critically involved in chronic inflammatory diseases (38–40). Matthews et al. have already impressively demonstrated that in *Il6*^-/-^ mice, hepatic insulin resistance and inflammation induced by a high-fat diet were worsened, pointing to a relationship between mitochondrial defects and cytokine dysregulation (41). Consistent with the protective role of IL-6-dependent STAT3 activation, hepatocyte-specific *Stat3*^-/-^ mice developed intensified liver injury and fibrosis after bile duct ligation than wild-type mice (42). It should be noted that chronic inflammatory conditions are often characterized by complex cytokine restructuring, whereby reduced IL-6 may be accompanied by compensatory upregulation of cytokines such as TNF-α, shifting the inflammatory balance toward more harmful signaling pathways despite apparently low IL-6 levels (43, 44). The reduced IL-6 levels may therefore indicate impaired liver regeneration or be linked to mitochondrial dysfunction, as mitochondrial pathways are involved in regulating cytokine production (45).

### 4.2 *Apoe*^-/-^ mice show an accelerated and more severe MASH phenotype

Compared to wild-type mice, *Apoe*^-/-^ mice developed a markedly more severe hepatic phenotype under Hf/Hf feeding, characterized by earlier and more pronounced steatosis, inflammation, and tissue injury. High-resolution non-destructive *ex vivo* MRI further revealed regional heterogeneity of hepatic steatosis across the parenchyma. This spatially resolved pattern is consistent with the histological findings of macrophage infiltration and early perisinusoidal collagen deposition, without evidence of advanced fibrosis.

In contrast to the prolonged time-course required for wild-type mice to develop inflammatory changes, *Apoe*^-/-^ mice exhibited robust inflammatory signatures starting already at 12 weeks of Hf/Hf feeding. The *Apoe*^-/-^ model displayed higher NAS scores, more severe alterations in cytokine profiles, pronounced oxidative stress, and gut barrier dysfunction compared to wild-type mice at any time point. These findings are mostly consistent with previous characterizations of *Apoe*^-/-^ mice feeding a Western diet for 7 weeks, leads to marked steatosis, macrophage infiltration, elevated hepatic cytokines (46). The mRNA expression analyses in *Apoe*^-/-^ mice revealed upregulation of several key inflammatory and stress-response genes. This transcriptional signature provides molecular insight into the pathways driving the severe phenotype in this model. Activating transcription factor 3 (ATF-3) is particularly interesting, as it is a stress-inducible transcription factor that is upregulated in response to diverse cellular stresses, including ER stress, oxidative stress, and inflammatory signals (47). In the liver, ATF-3 has context-dependent roles: it can act by inhibiting nuclear factor kappa-light-chain-enhancer of activated B-cells (NF-κB), as a negative feedback regulator of inflammatory signaling (48), but can also promote hepatocyte lipotoxicity and apoptosis under conditions of severe metabolic stress (49). The upregulation of *Atf3* in *Apoe*^-/-^ mice suggests that hepatocytes are experiencing significant cellular stress, likely from the combined burden of lipid overload, oxidative damage, and inflammatory signals. Higher mRNA levels of *Tnf*, *Ccl2* and prostaglandin-endoperoxide synthase 2 (*Ptgs2*, coding for COX-2) further demonstrate the inflammatory state in the liver. The immunohistochemical findings, like increased 3-NT, CD68, and IL-1β provide spatial confirmation of the molecular findings. The accumulation of 3-NT indicates severe oxidative/nitrosative stress, likely from the interaction of superoxide and nitric oxide to form peroxynitrite, a highly reactive species that damages proteins, lipids, and DNA (50). The increased CD68+ macrophages confirm robust Kupffer cell activation and monocyte recruitment, while IL-1β staining indicates inflammasome activation, a key feature of MASH pathogenesis (51, 52).

The accelerated disease progression in *Apoe*^-/-^ mice can be attributed to several factors. First, APOE deficiency causes severe hypercholesterolemia and altered lipoprotein metabolism, leading to hepatic cholesterol accumulation, which is highly lipotoxic and pro-inflammatory (53, 54). Second, APOE plays direct anti-inflammatory roles by modulating macrophage activation (55), suppressing NF-κB signaling (56), and inhibiting toll-like receptor (TLR)-induced cytokine production (57). Its absence therefore removes an important brake on inflammatory responses. Third, *Apoe*^-/-^ mice exhibit enhanced susceptibility to oxidative stress and endothelial dysfunction, creating a pro-inflammatory systemic milieu that promotes hepatic injury (58).

In the present study several qualitative differences between the two genotypes were revealed. In *Apoe*^-/-^ mice, a more robust systemic inflammation (elevated serum cytokines and AST), more severe histological inflammation (IHC for CD68 and IL-1β), in trend a more severe effects on oxidative stress markers (greater increases in 3-NT, with the same level of increased NQO1 activity and reductions in GPX activity) and impaired intestinal barrier function (elevated serum LPB and a greater impact on the expression of mRNA for colonic barrier markers) could be observed.

### 4.3 Oxidative stress and antioxidant dysregulation

The antioxidant imbalance, characterized by induction oFStress-response enzymes such as NQO1 alongside reduced scavenging capacity (GST, GPX), indicates an overwhelmed compensatory response to increased reactive oxygen species (ROS) production. Oxidative stress in MASH arises from multiple sources, including mitochondrial dysfunction, NADPH oxidase activation in Kupffer cells and hepatic stellate cells, and peroxisomal fatty acid oxidation (59, 60). The connection between oxidative stress and inflammation is bidirectional: ROS activate redox-sensitive transcription factors (e.g. NF-κB) that drive inflammatory cytokine expression, while inflammatory mediators (e.g. TNF-α, IL-1β) stimulate further ROS production through NADPH oxidase and mitochondrial pathways (61). The observation of increased oxidative stress markers (3-NT, NQO1, reduced GPX) and pro-inflammatory cytokines (IL-1α, CCL2, TNF-α) supports this connection. Quantitative MRS-based analysis of hepatic fatty acid composition provided additional metabolic context for the oxidative injury observed in *Apoe*^-/-^ mice. The depletion of PUFAs together with a relative increase in MUFAs is consistent with enhanced lipid peroxidation, given the high sensitivity of PUFAs to oxidative damage. Together with reduced GST and GPX activities, these findings suggest impaired antioxidant defenses and altered inflammatory lipid signaling. The more severe oxidative stress phenotype in *Apoe*^-/-^ mice may contribute to their enhanced disease progression and could explain their heightened inflammatory response.

### 4.4 Trace element homeostasis: copper dysregulation

Alterations in trace element homeostasis were observed in response to the Hf/Hf diet, with copper being particularly affected. Hepatic copper concentrations were reduced in both genotypes, whereas serum copper concentrations were increased in *Apoe*^-/-^ mice. These changes have mechanistic implications for MASH pathogenesis. Copper is an essential cofactor for several enzymes, including those involved in antioxidant defense (e.g. superoxide dismutase), mitochondrial respiration (e.g. cytochrome c oxidase), and iron metabolism (e.g. ceruloplasmin (CP)) (62). Reduced hepatic copper concentrations, as seen in the here presented mice models, were also found in human MASLD patients and have been associated with more pronounced hepatic steatosis, MASH, and other hallmarks of the metabolic syndrome (63). Dietary copper restriction in rodents also leads to the development of hepatic steatosis and insulin resistance (63, 64). A reduction in hepatic copper may impair copper-dependent antioxidant enzymes, contributing to the oxidative stress phenotype observed in the present study. Conversely, copper excess is associated with liver toxicity (e.g. Wilson disease), by promoting oxidative stress, activating nuclear factor erythroid 2-related factor 2 (NRF2) signaling and stimulating lipogenesis (65). The huge elevation oFSerum copper in *Apoe*^-/-^ mice, despite reduced hepatic copper, suggests either enhanced hepatic copper export, reduced hepatic uptake, or increased systemic copper mobilization from other tissues. The significant increase in CPO activity, reflecting higher CP levels and/or copper incorporation, provides a potential explanation for the elevated circulating copper observed in these mice. During hepatic inflammation and as part of acute-phase responses, CP levels and systemic copper levels can change (66, 67). Because CP binds and carries copper in plasma, increased CP synthesis or release (as an acute-phase response) can therefore elevate serum copper even if hepatocyte parenchymal copper is low or redistributed. Both clinical observations and mechanistic studies demonstrate that serum copper concentrations increase progressively along the spectrum of steatotic liver diseases, from ASimple steatotic liver to MASH, and continue to rise from cirrhosis to hepatocellular carcinoma (68, 69).

### 4.5 Oxylipin profiles and COX-derived inflammatory mediators

Oxylipin profiling revealed elevated COX-derived products in both wild-type and *Apoe*^-/-^ mice, with more severe alterations in the latter. Oxylipins are bioactive lipid mediators derived from polyunsaturated fatty acids (primarily arachidonic acid, eicosapentaenoic acid, and docosahexaenoic acid) through COX, LOX, and cytochrome P450 pathways. COX-derived products include PG and thromboxanes, which play diverse roles in inflammation, vascular function, and metabolic regulation (70, 71). The elevation of COX products in the here presented MASH models likely reflects increased COX-2 expression, which was confirmed at the mRNA level of *Ptgs2* in wild-type and *Apoe*^-/-^ mice. COX-2 is an inducible enzyme that is strongly upregulated by inflammatory stimuli and generates PG that can amplify inflammation through autocrine and paracrine signaling (71). PGD_2_, a major eicosanoid, promotes adipogenesis (71, 72) and activates TLR4/NF-κB signaling pathways to regulate the secretion of cytokines and chemokines (73). The marked oxylipin alterations, like PGD_2_, in *Apoe*^-/-^ mice mirror their more robust inflammatory phenotype and suggest that lipid mediator dysregulation contributes to disease severity. The parallel elevation of *Ptgs2* mRNA and COX products in *Apoe*^-/-^ mice, alongside increased TNF-α and CCL2, suggests coordinated activation of inflammatory signaling networks. COX-2 is a downstream target of NF-κB, which is also responsible for inducing TNF-α, IL-1β, and CCL2 expression (71, 73).

### 4.6 Gut–liver axis dysfunction: intestinal barrier impairment and endotoxemia

In the presented study, Hf/Hf diet-induced changes in mRNA expression of intestine barrier markers were confined to the distal colon, with no significant alterations in the ileum, highlights the region-specific vulnerability of the intestinal barrier to metabolic dietary stress. This differential response is consistent with multiple mechanisms that are specific to the colonic environment. First, the distal colon contains the highest microbial density in the gastrointestinal tract, and Hf/Hf diets induce dysbiosis characterized by loss of protective short-chain fatty acids-producing bacteria and secondary bile acid-converting species, changes that particularly impact on the colonic environment (74–76). Second, colonic mucus layer degradation as an early event in Hf/Hf feeding (75), increases epithelial exposure to pro-inflammatory microbial products. Third, the colon uniquely depends on microbially-produced secondary bile acids to maintain tight junction expression (77). Hf/Hf-induced depletion of these protective metabolites specifically compromises colonic barrier integrity (78, 79). In contrast, the ileum, despite facing direct exposure to dietary fat and primary bile acids, appears to have adaptive responses that preserve baseline tight junction expression, possibly through more rapid epithelial turnover or different temporal response kinetics (80). Indeed, Hamilton et al. demonstrated that ileal permeability increases are immediate but reversible, whereas large intestinal dysfunction is progressive and sustained (80). The here presented findings likely reflect this temporal pattern, capturing the sustained colonic response while ileal adaptations have already occurred. These results highlight the importance of region-specific therapeutic approaches targeting colonic barrier integrity in metabolic disease.

The mRNA analyses revealed striking genotype-specific responses to Hf/Hf diet in colonic barrier function. While both *Apoe*^-/-^ and wild-type mice showed reductions in *Ocln*, *Tjp1*, and *Muc2* expression, *Apoe*^-/-^ mice exhibited additional impairments not seen in wild-type controls, including reduced *Cldn1* and *C1galt1*. The opposite responses in *Cldn1* expression between genotypes may represent a critical determinant of barrier resilience. Wild-type mice showed modest *Cldn1* upregulation despite reductions in *Ocln* and *Tjp1*, suggesting a compensatory response. In contrast, *Apoe*^-/-^ mice showed downregulation across all major tight junction proteins, indicating failure of adaptive mechanisms. Beyond shared reductions in *Muc2* expression, *Apoe*^-/-^ mice showed a unique deficit in *C1galt1*, which is essential for mucin O-glycosylation. This represents a “double hit” to the mucus barrier: reduced mucin quantity combined with impaired mucin glycosylation quality, producing structurally deficient mucins that are more easily degraded by bacterial enzymes. Montrose et al. also investigated the role of fructose on the large intestine barrier. Showing a reduced mucus thickness (−20% after 1 week of high-fructose feeding) which was not due to reduced goblet cell number (81). This dissociation suggests that interventions targeting ER stress or glycosylation machinery may restore mucin production without requiring goblet cell regeneration. Important to acknowledge is that the observed changes in gut barrier are only based on mRNA analysis, intestinal permeability was not directly assessed in the present study. Nevertheless these molecular differences translated to functional consequences. Only *Apoe*^-/-^ mice showed elevated serum LBP concentrations, indicating metabolic endotoxemia.

These findings demonstrate that genetic factors affecting gut barrier resilience, including lipid metabolism genes like *Apoe*, may determine individual susceptibility to diet-induced metabolic endotoxemia and its downstream consequences. The *Apoe*^-/-^ model reveals how failure of compensatory mechanisms can amplify dietary stress into systemic metabolic disease through the gut–liver axis.

### 4.7 Proposed model for MASLD-to-MASH progression under dietary intervention

It is challenging to synthesize the findings presented here with the existing literature, because the huge heterogeneity of dietary models used across studies. Published MASH models employ diverse dietary manipulations including Western diets, high-fat plus fructose/sucrose combinations, high-fat/high-cholesterol/high-sugar supplements and more variations, each with different fat percentages, sugar sources, and micronutrient compositions. These compositional differences substantially affect the kinetics and severity of disease progression. Despite this heterogeneity, common mechanistic themes emerge across models that allow us to propose a conceptual framework for MASH progression, while acknowledging that specific timelines are model- and diet-dependent.

**Phase 1 – Steatosis (8–16 weeks in wild-type C57BL/6J)** (82–85)

- Hepatic steatosis because of increased lipogenesis, impaired fatty acid oxidation, and enhanced dietary lipid uptake.
- Initial oxidative stress develops from mitochondrial overload and peroxisomal fatty acid oxidation.
- Gut microbiota alterations begin, with early changes in barrier function.
- NLRP3 inflammasome is activated in the liver.
- Systemic inflammatory responses remain largely absent due to the presence of compensatory anti-inflammatory mechanisms.

**Phase 2 – Transition (16–24 weeks in wild-type C57BL/6J)** (46, 86, 87)

- Sustained lipotoxicity beginning of hepatocyte apoptosis.
- Trace element homeostasis starts to shift.
- Oxidative stress intensifies, with depletion of antioxidant defenses and compensatory upregulation of stress-response enzymes.
- Gut barrier dysfunction progresses, allowing bacterial product translocation.
- Hepatocyte stress signals (DAMPs) and gut-derived PAMPs (LPS) activate innate immune receptors.

**Phase 3 – Manifested steatohepatitis (≥24 weeks in wild-type C57BL/6J; 7–12 weeks in *Apoe*^-/-^)** (9, 46, 86, 88–90)

- Severe inflammatory cascade activation: NF-κB-driven cytokine/chemokine expression.
- Macrophage recruitment and M1 polarization.
- COX-2 induction and oxylipin dysregulation, creating lipid mediator-driven inflammatory amplification.
- Cellular stress response activation.
- Severe oxidative/nitrosative stress.
- Systemic inflammatory spillover and more severe hepatocellular injury.

### 4.8 Limitations

Several limitations of the presented study should be acknowledged. First, the gut barrier assessment was based primarily on mRNA analysis. Histological analyses to evaluate epithelial or mucus layer integrity and detailed microbiome profiling, which would provide insight into the specific bacterial taxa and metabolic pathways contributing to gut barrier dysfunction and endotoxemia were not performed. Furthermore, direct functional measurements of intestinal permeability (e.g., FITC-dextran assay) were not performed. Future studies integrating molecular, histological, and functional readouts will provide a more comprehensive characterization of intestinal barrier integrity in this model. Second, mechanistic studies underlining the findings for trace elements are also reasonable: What are the specific molecular mechanisms linking copper dysregulation to oxidative stress and inflammation using the herein established model? Third, future studies should also investigate sex differences in MASH progression, as the here presented study used only male mice. Epidemiological data show that men are more frequently affected by MASLD than women (91). Gender-specific differences in the course of the disease have also been described in various animal models, although with contradictory results (92, 93).

Finally, intervention studies testing therapeutic strategies targeting the pathways identified in this study would be valuable. Potential approaches include: (i) antioxidant therapies to address oxidative stress, (ii) trace element modulation, (iii) COX-2 inhibitors or LOX modulators, shifting oxylipin profiles in a more anti-inflammatory direction.

## 5 Conclusions

The comparative longitudinal study of wild-type and *Apoe*^-/-^ mice fed a Hf/Hf diet provides important insights into the temporal progression and mechanistic drivers of MASH. It has been demonstrated that steatosis and inflammation are temporally dissociated, with inflammation emerging only after prolonged steatosis (24 weeks in wild-type), and that *Apoe*^-/-^ dramatically accelerates and intensifies all aspects of MASH pathogenesis. The disease progression involves a complex interplay of oxidative stress, trace element dysregulation, oxylipin imbalance, and gut–liver axis dysfunction, with these pathways converging to trigger the inflammatory cascade that defines steatohepatitis. The results presented in this study highlight multiple potential therapeutic targets and underscore the importance of model selection in preclinical MASH research. Understanding the mechanisms that drive the transition from benign steatosis to inflammatory steatohepatitis is critical for developing effective therapies to halt or reverse this increasingly prevalent and morbid disease.

## Data Availability

The original contributions presented in the study are publicly available. This data can be found here: Zenodo, 10.5281/zenodo.21277289.

## Supplementary material

Supplementary Figure S1 mRNA measurements of markers of intestinal barrier integrity after high-fat/high-fructose (Hf/Hf) diet. Duration of diet 24 weeks in wild-type and 12 weeks in *Apoe*^-/-^. Relative mRNA levels were measured in ileum for **(A)** claudin 1 (*Cldn1*), **(B)** claudin 3 (*Cldn3*), **(C)** occludin *(Ocln)*, **(D)** tight junction protein 1 (*Tjp1*), **(E)** mucin 2 (*Muc2*), **(F)** core 1 synthase, glycoprotein-N-acetylgalactosamine 3-beta-galactosyltransferase, 1 (*C1galt1*). Data are displayed as log_2_ (fold change (FC)) relative to control. Boxes represent the interquartile range (25th–75th percentiles), with the median indicated as center line. Whiskers extend from the minimum to the maximum value. Individual data points are shown. Sample sizes varied between analyses due to assay-specific sample availability and exclusion of statistical outliers as described in the statistical analysis section. Statistical analysis was performed using two-way ANOVA followed by Holm-Sidak *post-hoc* test. **p* < 0.05, ***p* < 0.01, ****p* < 0.001 vs. control; § vs. 24 weeks.

Supplementary Table 1 Composition of the experimental diets used in this study. Animals were fed either a control diet or a high-fat/high-fructose (Hf/Hf) diet.

Supplementary Table 2 Sequences of the RT-qPCR primers used in the study. The forward and reverse primers are located in different exons.

Supplementary Table 3 LC-gradient-Nexera X2 UHPLC system (Shimadzu Corporation, Kyoto, Japan). The solvents consist of (A) water, methanol and formic acid (89.95:9.95:0.1 v/v) and (B) methanol and formic acid (99.9:0.1 v/v).

Supplementary Table 4 Global MS/MS parameters for the QTRAP 7500 mass spectrometer (AB Sciex, Darmstadt, Germany).

Supplementary Table 5 Analyte-specific MS/MS parameters for the determination of oxylipins using scheduled MRM mode on the QTRAP 7500 system. Reconstitution of evaporated solid phase extracted samples in 100 µl (giving quantification limits (LLOQs) in sample), injection of 10 µl into UPLC-MS/MS system (giving LLOQs on column). The LLOQ was defined as the lowest concentration level on the calibration curve at which all three independently prepared samples yielded a signal-to-noise ratio of > 5.
