# Supplementary figures and images for: Superior inflammatory response and MASH progression in Apoe-/- mice compared to wild-type mice: a comprehensive time-course analysis
Source: Front Immunol. 2026 Jul 17;17:1895809. doi: 10.3389/fimmu.2026.1895809 (PMC13423675; doi:10.3389/fimmu.2026.1895809)

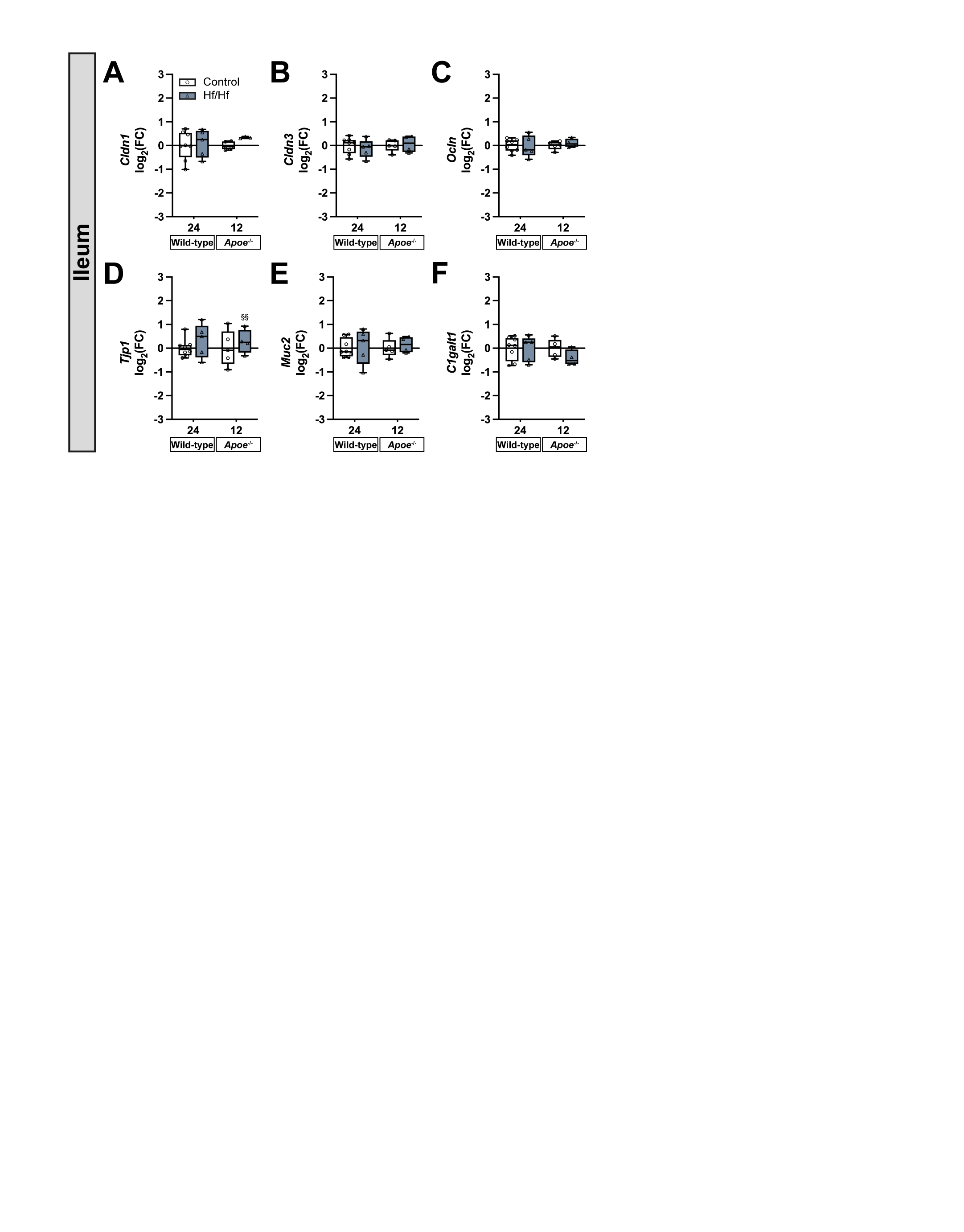

Supplement: Supplementary Figure S1 — mRNA measurements of markers of intestinal barrier integrity after high-fat/high-fructose (Hf/Hf) diet. Duration of diet 24 weeks in wild-type and 12 weeks in Apoe-/-. Relative mRNA levels were measured in ileum for (A) claudin 1 (Cldn1), (B) claudin 3 (Cldn3), (C) occludin (Ocln), (D) tight junction protein 1 (Tjp1), (E) mucin 2 (Muc2), (F) core 1 synthase, glycoprotein-N-acetylgalactosamine 3-beta-galactosyltransferase, 1 (C1galt1). Data are displayed as log2 (fold change (FC)) relative to control. Boxes represent the interquartile range (25th–75th percentiles), with the median indicated as center line. Whiskers extend from the minimum to the maximum value. Individual data points are shown. Sample sizes varied between analyses due to assay-specific sample availability and exclusion oFStatistical outliers as described in the statistical analysis section. Statistical analysis was performed using two-way ANOVA followed by Holm-Sidak post-hoc test. *p < 0.05, **p < 0.01, ***p < 0.001 vs. control; § vs. 24 weeks. [file Image1.tif]
